# Modeling Nursing Home Harms From COVID-19 Staff Furlough Policies

**DOI:** 10.1001/jamanetworkopen.2024.29613

**Published:** 2024-08-19

**Authors:** Sarah M. Bartsch, Colleen Weatherwax, Bruce Leff, Michael R. Wasserman, Raveena D. Singh, Kavya Velmurugan, Danielle C. John, Kevin L. Chin, Kelly J. O’Shea, Gabrielle M. Gussin, Marie F. Martinez, Jessie L. Heneghan, Sheryl A. Scannell, Tej D. Shah, Susan S. Huang, Bruce Y. Lee

**Affiliations:** 1 Center for Advanced Technology and Communication in Health, City University of New York Graduate School of Public Health and Health Policy, New York; 2 Public Health Informatics, Computational, and Operations Research, City University of New York Graduate School of Public Health and Health Policy, New York; 3 Artificial Intelligence, Modeling, and Informatics for Nutrition Guidance and Systems Center, City University of New York Graduate School of Public Health and Health Policy, New York; 4 Division of Geriatric Medicine and Gerontology, The Center for Transformative Geriatric Research, Johns Hopkins School of Medicine, Baltimore, Maryland; 5 California Association of Long Term Care Medicine, Santa Clarita; 6 Division of Infectious Diseases, Department of Medicine, University of California Irvine School of Medicine, Irvine; 7 New York City Pandemic Response Institute, New York

## Abstract

**Importance:**

Current guidance to furlough health care staff with mild COVID-19 illness may prevent the spread of COVID-19 but may worsen nursing home staffing shortages as well as health outcomes that are unrelated to COVID-19.

**Objective:**

To compare COVID-19–related with non–COVID-19–related harms associated with allowing staff who are mildly ill with COVID-19 to work while masked.

**Design, Setting, and Participants:**

This modeling study, conducted from November 2023 to June 2024, used an agent-based model representing a 100-bed nursing home and its residents, staff, and their interactions; care tasks; and resident and staff health outcomes to simulate the impact of different COVID-19 furlough policies over 1 postpandemic year.

**Exposures:**

Simulating increasing proportions of staff who are mildly ill and are allowed to work while wearing N95 respirators under various vaccination coverage, SARS-CoV-2 transmissibility and severity, and masking adherence.

**Main Outcomes and Measures:**

The main outcomes were staff and resident COVID-19 cases, staff furlough days, missed care tasks, nursing home resident hospitalizations (related and unrelated to COVID-19), deaths, and costs.

**Results:**

In the absence of SARS-CoV-2 infection in the study’s 100-bed agent-based model, nursing home understaffing resulted in an annual mean (SD) 93.7 (0.7) missed care tasks daily (22.1%), 38.0 (7.6) resident hospitalizations (5.2%), 4.6 (2.2) deaths (0.6%), and 39.7 (19.8) quality-adjusted life years lost from non–COVID-19–related harms, costing $1 071 950 ($217 200) from the Centers for Medicare & Medicaid Services (CMS) perspective and $1 112 800 ($225 450) from the societal perspective. Under the SARS-CoV-2 Omicron variant conditions from 2023 to 2024, furloughing all staff who tested positive for SARS-CoV-2 was associated with a mean (SD) 326.5 (69.1) annual furlough days and 649.5 (95% CI, 593.4-705.6) additional missed care tasks, resulting in 4.3 (95% CI, 2.9-5.9) non–COVID-19–related resident hospitalizations and 0.7 (95% CI, 0.2-1.1) deaths, costing an additional $247 090 (95% CI, $203 160-$291 020) from the CMS perspective and $405 250 (95% CI, $358 550-$451 950) from the societal perspective. Allowing 75% of staff who were mildly ill to work while masked was associated with 5 additional staff and 5 additional resident COVID-19 cases without added COVID-19–related hospitalizations but mitigated staffing shortages, with 475.9 additional care tasks being performed annually, 3.5 fewer non–COVID-19–related hospitalizations, and 0.4 fewer non–COVID-19–related deaths. Allowing staff who were mildly ill to work ultimately saved an annual mean $85 470 (95% CI, $41 210-$129 730) from the CMS perspective and $134 450 (95% CI, $86 370-$182 540) from the societal perspective. These results were robust to increased vaccination coverage, increased nursing home transmission, increased importation of COVID-19 from the community, and failure to mask while working ill.

**Conclusion and Relevance:**

In this modeling study of staff COVID-19 furlough policies, allowing nursing home staff to work with mild COVID-19 illness was associated with fewer resident harms from staffing shortages and missed care tasks than harms from increased COVID-19 transmission, ultimately saving substantial direct medical and societal costs.

## Key Points

**Question:**

What is the tradeoff between COVID-19–related harms and non–COVID-19–related harms when allowing nursing home staff with mild COVID-19 to work while masked?

**Findings:**

In this modeling study that used an agent-based model of a 100-bed nursing home, understaffing (without SARS-CoV-2 infections) was associated with missed tasks, resident hospitalizations, and deaths, costing an annual $1 071 950, and furloughing staff testing positive for SARS-CoV-2 infection was associated with additional missed tasks, non–COVID-19 hospitalizations, and deaths, costing an additional $247 090 from the perspective of the Centers for Medicare & Medicaid Services (CMS). However, allowing 75% of nursing home staff who were mildly ill to work averted most of these non–COVID-19 harms, saving $85 470 from the CMS perspective without worsening staff or resident COVID-19 hospitalizations.

**Meaning:**

The findings of this study suggest that allowing nursing home staff who were mildly ill with COVID-19 to work while masked was associated with less harm from alleviated missed tasks, outweighing increasing harm from COVID-19 transmission.

## Teaser

This modeling study examines whether furloughing nursing home staff with mild COVID-19, which may prevent virus transmission, might worsen staffing shortages and non–COVID-19–related health outcomes.

## Introduction

Currently, nursing home staff who test positive for SARS-CoV-2 are furloughed for up to 10 days to reduce spread. However, given understaffing strains, furloughs may have detrimental consequences on resident care and burnout for remaining staff. Nursing home staffing shortages preceded the COVID-19 pandemic.^1,2,3,4,5,6,7,8^ From 2017 to 2018, 91% of US nursing homes were short staffed for registered nurses (RNs) more than 40% of the time.^3^ During the COVID-19 pandemic,^9^ staff shortages negatively impacted care activities (eg, feeding, hydration, mobility support, bathing, and medication administration), resulting in adverse outcomes (eg, bedsores, dehydration, and weight loss).^9,10,11^ Thus, it is important to understand the potential tradeoffs in furloughing staff to protect residents and other staff from COVID-19 vs not meeting residents’ needs due to staff absences. Therefore, we adapted a computational agent-based model of a nursing home to estimate the clinical and economic impacts of different COVID-19 staff furlough policies.

## Methods

### Agent-Based Model Overview and Mixing

In this modeling study simulating 1 postpandemic year, we used a previously described agent-based model (Python, version 3.9 [Python Software Foundation]),^12,13^ representing a typical 100-resident nursing home with staff; resident and staff interactions; and SARS-CoV-2 testing, transmission, and resultant health and economic outcomes for residents and staff.^14,15,16,17,18,19,20^ The nursing home consisted of 50 double rooms, grouped in 10-room hallways or housing areas, to which clinical staff were assigned daily. We represented resident-facing staff providing routine care (eg, certified nursing assistants [CNAs], RNs, and environmental cleaning staff), resident-facing staff providing specialty care (eg, physical, occupational, and speech therapy), and non–resident-facing staff (eg, administration, laundry, and kitchen). Table 1 shows select model parameters, values, and sources.^21,22,23,24,27,28,29,30,31,32,33,34,35,36^ (All values are provided in eTables 1-6 in Supplement 1.) This study met criteria for human participant exemption, as determined by the University of California Irvine institutional review board; thus, informed consent was waived. This study followed the Consolidated Health Economic Evaluation Reporting Standards (CHEERS) reporting guideline as well as the Modeling Infectious Diseases in Healthcare Network (MInD-Healthcare) framework.

**Table 1.** Key Model Input Parameters, Values, and Sources

Parameter	Value	Source
Probability of transmission given effective contact, %	3.0	Calibrated^a^
Staff-to-staff reduction in mixing and transmission for other precautions (eg, hand hygiene, physical distancing), %	70.0	Calibrated^a^
Vaccination coverage with current annual vaccine, %^b^		
Staff	22.9	CDC^21^
Residents	38.1	CDC^21^
Vaccine efficacy against infection (0-3 mo), %	65.0	Assumption^c^
Vaccine efficacy against infection (ending protection after 6 mo), %	32.5	Assumption^d^
Vaccine efficacy against hospitalization, median (range), %	76.1 (62.3-84.8)	Hansen et al^22^
N95 respirator efficacy, %	99.0	Lindsley et al^23^
N95 respirator compliance (included unmasked mealtimes), %	75.0	Kendra et al^24^
Staff who were infectious being tested for SARS-CoV-2, %	50.0	Gussin et al^25^ and Shang et al^26^
Residents who were infectious reported or showed COVID-19 symptoms and tested for SARS-CoV-2, %	30.0	Expert opinion^a^
Staff shift length (excluding breaks), h	7.0	Assumption^1^
Time per shift spent on tasks, %	80.0	Expert opinion^a^
Time needed for tasks of daily living, per resident, per d, min		
Exercising or moving residents who were nonbed bound	35.0	10 NH survey^e^
Exercising or moving residents who were bed bound	28.0	10 NH survey^e^
Feeding and hydrating^f^	36.0	10 NH survey^e^
Giving medications^g^	30.0	10 NH survey^e^
Turning (residents who were bed bound)^h^	10.0	10 NH survey^e^
Providing toileting assistance or other hygiene-related tasks such as wound care^i^^,^^j^	38.0	10 NH survey^e^
RN time spent on medication-related tasks, %	70.0	Calibrated^k^
Time until harm if task was not completed, median (range), d		
Exercising or moving residents	4.0 (3.0-5.0)	Expert opinion^27^
Turning (residents who were bed bound)	2.0 (1.0-3.0)	Expert opinion^28,29^
Feeding and hydrating	2.0 (1.0-3.0)	Expert opinion^30,31^
Giving essential medications	2.5 (1.0-4.0)	Expert opinion^32,33^
Providing toileting assistance or other hygiene-related tasks	2.0 (1.0-3.0)	Expert opinion^34,35,36^

Abbreviations: CDC, Centers for Disease Control and Prevention; NH, nursing home; RN, registered nurse.

^a^ Descriptions of the calibration methods and synthesis of data by experts (B.L., M.R.W., and S.S.H.) are provided in the eMethods in Supplement 1.

^b^ Vaccination coverage of residents and staff reported through January 7, 2024.

^c^ Assumed similar to influenza vaccine efficacy.

^d^ Assumed 50% reduction.

^e^ Obtained from a survey of 10 Southern California nursing homes, conducted by R.D.S. and S.S.H., that provided summary values based on staffing records and direct observation (eMethods in Supplement 1).

^f^ Observed feeding and hydration tasks on average lasted 12 minutes and occurred 3 times (3 meals) per day.

^g^ Observed medication tasks on average lasted 15 minutes and occurred 3 times per day (including assessment time); adjusted to 2 times per day (excluding night shift).

^h^ Observed turning tasks on average lasted 5 minutes and occurred 3 times per day; adjusted to 2 times per day (excluding night shift).

^i^ Assumed that bathing residents occurred 3 times per week.

^j^ Observed toileting and hygiene tasks on average lasted 6 minutes and occurred 3 times per day; wound care tasks on average lasted 10 minutes and occurred 2 times per day.

^k^ Calculated using the proportion of time needed for resident medication tasks compared with other tasks and number of RN hours available (eMethods in Supplement 1).

**Figure 1.**
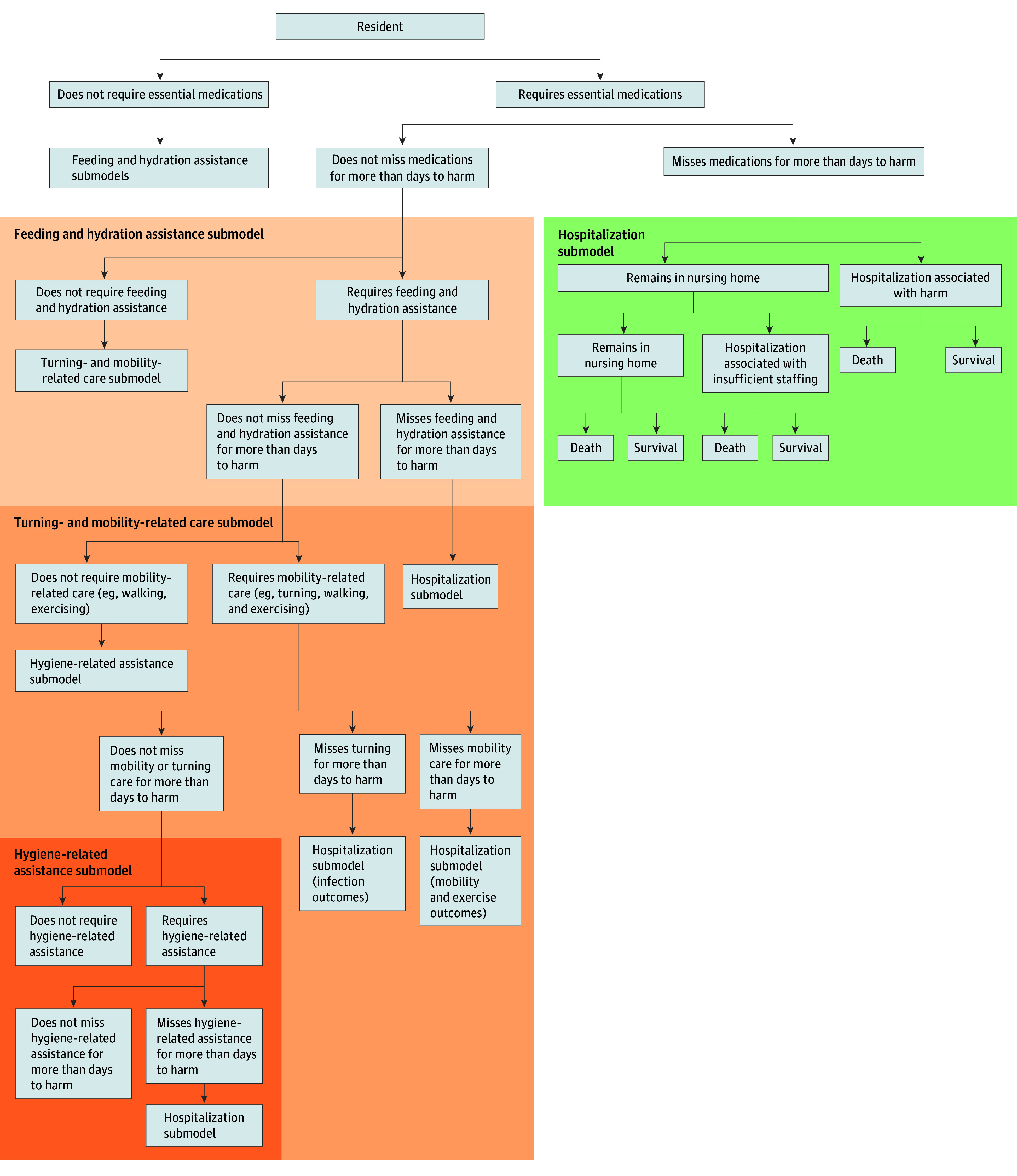
Resident Tasks Needed and Potential Harm if Not Received

Each resident had a set of characteristics (Figure 1) designating their daily care needs (eg, mobile support, feeding assistance, turning or mobility assistance, and receiving medications). The model advanced in discrete, 1-day time steps for 1 year. Each day, staff and residents interacted (eFigure 1 in Supplement 1) until leaving the nursing home (length-of-stay elapsed, hospitalized, died, or left job). A resident’s interactions with staff or other residents were greater within their housing area, designated social groups and connections, and assigned staff. Staff interactions depended on their job type and assignments.

### Staff Roles and Activities

Resident-facing staff were assigned to a hallway or housing area, with staff interacting more with residents in an assigned area for continuity of care. We represented clinical care staff (CNAs, licensed practical nurses, and RNs) performing different tasks for residents. We modeled day and evening shifts when most tasks are performed.

We represented 4 major categories of tasks: feeding and hydrating; administering essential medications; turning, exercising, and moving residents; and hygiene-related tasks (eg, toileting assistance, bathing and showering, dressing, brushing teeth, and wound and device care). These tasks were performed daily based on residents’ needs (Figure 1). Staff covered tasks of furloughed staff if they had time (eg, a CNA fed residents assigned to a furloughed CNA). If staff did not have time to complete tasks (ie, total hours worked was less than hours needed), they dropped tasks for randomly selected residents. Staff dropped tasks until their task list could be done within their hours worked. Staff were less likely to drop critical tasks (eTable 6 in Supplement 1) but had a chance of dropping each task, given residents’ variable needs and requests (eg, incontinence episodes causing CNAs to prioritize cleaning before feeding).

### Resident Health Outcomes

Residents could experience the following 4 types of harm based on tasks not performed^37,38,39^ (Figure 1) and task-specific days to harm (Table 1): (1) harm from lack of mobility support (eg, falls, weakness from lack of exercise); (2) harm from missed feeding or hydration (eg, dehydration, failure to thrive, or weakness); (3) harm from missed essential medications (eg, diuretic, cardiac, psychiatric, or diabetic medications); and (4) infections (eg, pneumonia, urinary tract infections) from missed turning, hygiene, or wound or pressure ulcer care. Residents experiencing harm outcomes had a harm-specific probability of hospitalization. Hospitalized residents had an outcome-specific length of stay and probability of in-hospital mortality at the end of their stay. Residents not hospitalized had a daily probability of in-nursing home mortality due to harm. Residents experiencing harm were always hospitalized when there were fewer than 50 routine staff available (ie, insufficient staffing).

### SARS-CoV-2 Transmission

eFigure 1 in Supplement 1 shows previously described^12,13^ mutually exclusive SARS-CoV-2 states that each resident and staff could be in and how they moved through them. Each day, residents and staff interacted with each other, and a person who was infected could transmit SARS-CoV-2 to a person who was susceptible (eMethods in Supplement 1). After recovering, residents and staff had natural immunity against infection (waned over time) (eMethods in Supplement 1) and hospitalization (long lasting^40^).

### Resident and Staff COVID-19–Related Health Outcomes

Residents and staff who were symptomatic started with mild infection with a probability of progressing to severe disease requiring hospitalization^12,13^ and had a probability of being treated with antiviral medications. Each hospitalized individual had a length of stay and probability of COVID-19–related mortality at the end of their stay. Residents who survived with a length of stay of 10 or fewer days returned to the nursing home (ie, their bed was held); those with longer lengths of stays left the model.

### SARS-CoV-2 Testing, Isolation, and Furloughs

Staff were tested when disclosing symptoms or when overt symptoms were noted, as previously described^12,13^ (eFigure 2 in Supplement 1), assuming that 50% of those infectious were tested.^25,26^ Staff awaiting test results wore N95 respirators and continued working. Staff who tested positive were furloughed for 7 or more days from test positivity or until they tested negative according to national guidance.^41^ When staff who were mildly ill were allowed to work, they wore N95 respirators for their infectious period duration.

Residents who were symptomatic were isolated while awaiting results. Residents who tested positive were isolated for 10 days and their roommates quarantined for 10 days. Resident isolation and quarantine required an N95 respirator, eye protection, gown, and glove use by staff.

### Costs and Economic Outcomes

Each person accrued relevant costs and health effects as they moved through the model. Residents with multiple negative health outcomes from missed tasks incurred only the first outcome by days to harm. The model generated health effects and costs (in 2024 values) from the Centers for Medicare & Medicaid Services (CMS), total third-party payers, and the societal perspective (eMethods in Supplement 1).

### Statistical Analysis

Our first set of scenarios assumed all staff who tested positive for SARS-CoV-2 were furloughed. The second set of scenarios allowed 25% to 75% of staff who were mildly symptomatic to work with N95 respirators. Sensitivity analyses varied the annual vaccination coverage of residents (38% to 76%) and staff (23% to 46%), transmission probability (3% to 10%), community COVID-19 risk (1.3 times baseline values), COVID-19 severity (up to 3 times the baseline hospitalization risk), and staff who were infected and underwent testing (35% to 65%). Additional scenarios assumed the staff wore surgical masks or no masks while results were pending or while working mildly ill.

Experiments consisted of performing 200 trials. Statistical analyses, including calculations of SDs and 95% CIs, were done using Python, version 3.9. We conducted the study from November 2023 to June 2024.

## Results

### Baseline Nursing Home Staffing Levels in the Absence of SARS-CoV-2

Within the 100-bed nursing home agent-based model, baseline simulations using actual staffing data showed insufficient staffing to cover resident care tasks even without SARS-CoV-2 infection. The 4 task categories required a mean 2.4 hours of care per resident-day. However, current staffing levels permitted only 1.7 hours per resident. Thus, a mean (SD) 93.7 (0.7) of 424.0 daily resident tasks (22.1%) were not completed. This translated to a mean (SD) 34 184.7 (247.2) of 154 760.0 tasks missed each year (34 185.7 per 100 person-years). Annually, missed tasks were associated with a mean (SD) 38.0 (7.6) hospitalizations (5.2% of residents), 4.6 (2.2) deaths (0.6% of residents), and 39.7 (19.8) quality-adjusted life-years lost, costing $1 071 950 ($217 200) from the CMS perspective and $1 112 800 ($225 450) from the societal perspective per 100-bed nursing home.

### Adding SARS-CoV-2 and Furloughing All Staff Who Tested Positive

**Figure 2.**
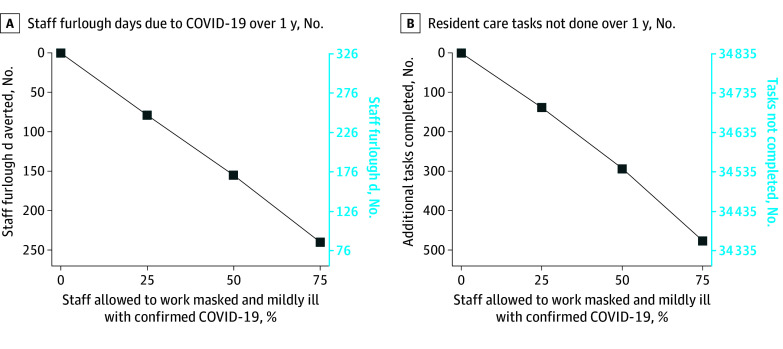
Estimated Impact of Nursing Home Staff Furlough Policies Compared With Current Understaffing Levels on Nursing Home Operational Outcomes Simulated nursing home staff furlough policies allowed different proportions of staff who were mildly ill to work while wearing N95 respirators. Note difference in axis scales across panels.

**Figure 3.**
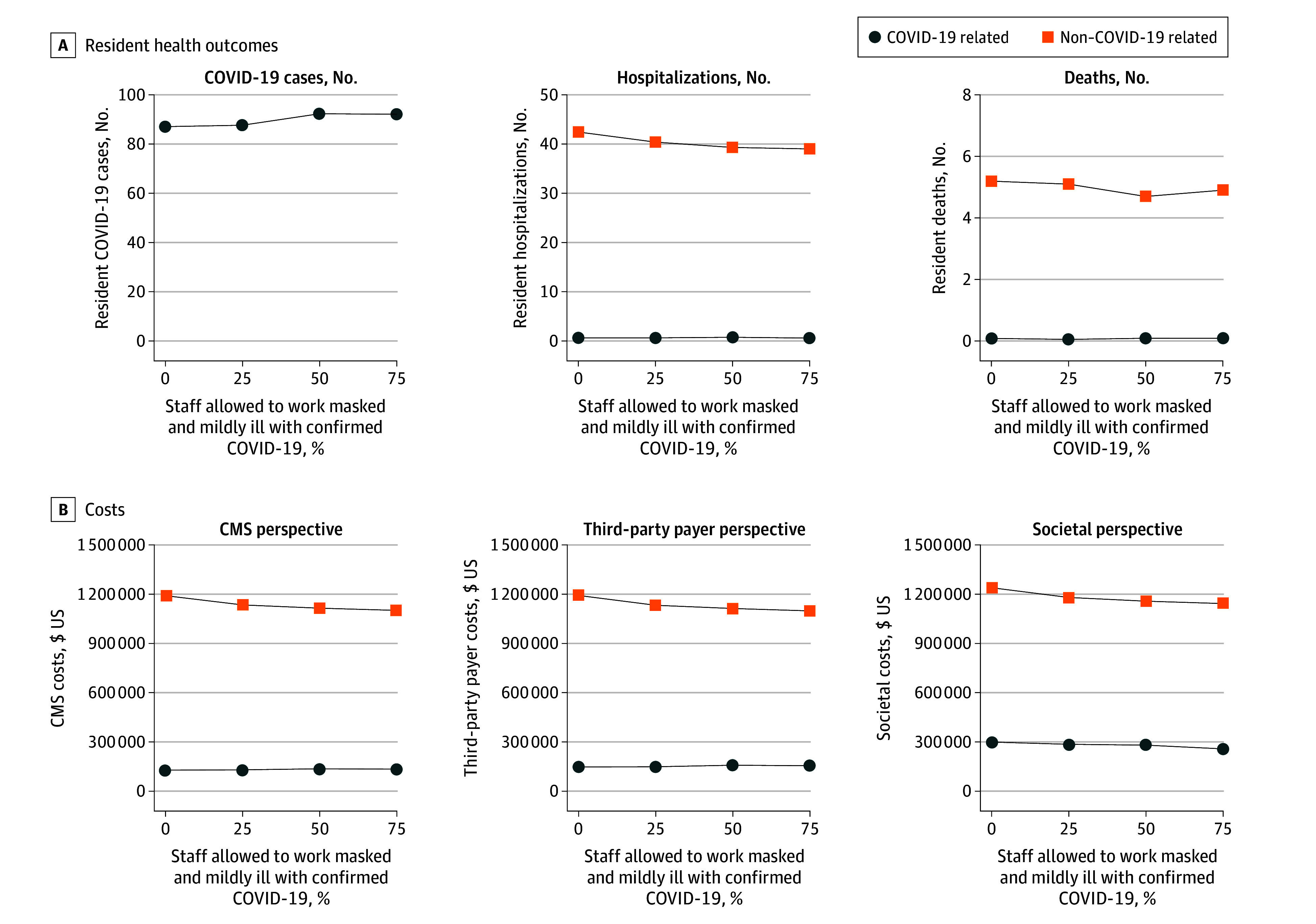
Estimated Impact of Nursing Home Staff Furlough Policies Compared With Current Staffing Levels on COVID-19–Related and Non–COVID-19–Related Resident Outcomes and Costs Simulated nursing home staff furlough policies allowed different proportions of staff who were mildly ill to work while wearing N95 respirators. Note the difference in axis scales across panels. The third-party payer included combined direct medical costs for residents and staff. CMS indicates Centers for Medicare & Medicaid Services.

Introducing SARS-CoV-2 and furloughing all staff who tested positive for the Omicron variant during the simulated year for 7 days or more (corresponding to 0% of staff allowed to work mildly ill in Figure 2 and Figure 3) worsened understaffing. Annually, a mean (SD) 113.2 (20.9) staff COVID-19 cases occurred (47.3% community onset), incurring 326.5 (69.1) furlough days. Even though other staff could cover some tasks, furloughs were associated with an additional mean 649.5 (95% CI, 593.4-705.6) missed tasks annually (1.8 tasks per day). For residents, additional missed tasks were associated with a mean 4.3 (95% CI, 2.9-5.9) additional non–COVID-19–related hospitalizations (4.3 per 100 person-years; 5.8% of residents) and 0.7 (95% CI, 0.2-1.1) non–COVID-19–related deaths (0.7% of residents) associated with COVID-19–related furloughs. There was a mean (SD) 87.1 (26.3) COVID-19 cases, 0.7 (0.8) COVID-19 hospitalizations, and 0.1 (0.2) deaths among residents. Together, COVID-19–related and non–COVID-19–related outcomes associated with COVID-19 staff furloughs cost an additional mean $247 090 (95% CI, $203 160-$291 020) from the CMS perspective and $405 250 (95% CI, $358 550-$451 950) from the societal perspective. However, across all perspectives, costs associated with non–COVID-19–related harms outweighed costs associated with COVID-19, ranging from $1 236 490 in total non–COVID-19–related costs compared with $298 100 in total COVID-19–related costs (4.1 times higher) to $1 191 390 in total non–COVID-19–related costs compared with $127 680 in total COVID-19–related costs (9.3 times higher).

### Allowing Staff With Mild COVID-19 Illness to Work While Masked

Allowing increasing proportions of staff who were mildly ill to work while wearing N95 respirators resulted in fewer furlough days, more completed tasks, more COVID-19–related and fewer non–COVID-19–related (missed task–related) health outcomes, and lower costs (Figure 2 and Figure 3). Non–COVID-19–related outcomes and costs consistently outweighed COVID-19 outcomes when staff who were mildly ill worked while masked (Figure 3 and Table 2).

**Table 2.** Annual COVID-19–Related and Non–COVID-19–Related Health Outcomes and Costs Comparing Staff Furloughs in a 100-Bed Nursing Home^a^

Parameter	Staffing outcome		Outcomes when allowing staff who were mildly ill to work compared with furloughing all staff^b^													
			COVID-19–related outcomes on staff			COVID-19–related outcomes on residents^c^					Non–COVID-19–related outcomes on residents^c^					
	Averted furlough, d	Additional tasks completed^c^	Additional cases	Additional hospitalizations	Additional cases		Additional hospitalizations	Additional deaths	Additional costs, $		Hospitalizations averted	Deaths averted	Cost savings, $		
									CMS perspective^d^	Societal perspective^d^			CMS perspective	Societal perspective
Current Omicron conditions and vaccination coverage	240.0 (229.6 to 250.4)	475.9 (418.1 to 533.7)	5.0 (0.5 to 9.4)	0.1 (−0.1 to 0.2)	5.0 (−0.6 to 10.6)		−0.1 (−0.2 to 0.1)	0 (0 to 0.1)	5 950 (−3612 to 15 512)	−39 606 (−56 256 to −22 955)	3.5 (1.9 to 5.0)	0.4 (−0.1 to 0.8)	91 422 (48 290 to −134 554)	94 849 (50 080 to 139 618)
Doubling vaccination coverage	204.5 (193.8 to 215.3)	416.5 (359.8 to 473.3)	1.8 (−2.8 to 6.4)	0 (−0.1 to 0.2)	3.6 (−1.6 to 8.7)		0 (−0.1 to 0.2)	0 (−0.1 to 0)	4648 (−4159 to 13 455)	−37 053 (−52 714 to −21 392)	2.3 (0.9 to 3.8)	0.3 (−0.1 to 0.7)	53 603 (12 195 to 95 011)	55 490 (12 520 to 98 460)
Transmission probability, 0.1	498.2 (471.8 to 524.5)	984.1 (902.8 to 1065.4)	0.4 (−12.0 to 12.7)	0.1 (−0.1 to 0.3)	−0.2 (−13.6 to −13.2)		0 (−0.3 to 0.2)	0 (0 to 0.1)	5683 (−14 616 to 25 982)	−94 341 (−13 196 to −56 719)	6.9 (5.3 to 8.5)	0.7 (0.2 to 1.1)	180 949 (135 818 to 226 080)	187 675 (140 842 to 234 508)
Community infection risk, 1.3× baseline values	255.8 (245.2 to 266.3)	495.3 (436.4 to 554.1)	2.7 (−1.7 to 7.1)	0 (−0.1 to 0.1)	3.4 (−2.3 to 9.1)		−0.2 (−0.3 to 0)	0 (−0.1 to 0)	3746 (−5889 to 13 381)	−46 473 (−63 270 to −29 675)	2.7 (1.1 to 4.3)	0.3 (−0.1 to −0.8)	80 821 (34 878 to 126 764)	83 483 (35 802 to 131 164)
COVID-19 hospitalization risk, 3× baseline values	236.4 (225.8 to 247.0)	632.4 (572.6 to 692.1)	2.1 (−2.6 to 7.1)	−0.1 (−0.3 to 0.2)	4.3 (−1.2 to 9.7)		0.2 (−0.2 to 0.5)	0 (−0.1 to 0.1)	13 121 (465 to 25 777)	−31 942 (−52 042 to −11 841)	1.3 (−0.2 to 2.9)	0.4 (0 to 0.9)	38 395 (−4883 to 81 673)	39 608 (−5336 to 84 552)
Staff wearing surgical masks or N95 respirators	244.1 (234.1 to 254.0)	503.0 (442.7 to 563.2)	2.4 (−2.2 to 6.9)	0.1 (0 to 0.2)	5.0 (−0.7 to 10.8)		0.1 (−0.1 to 0.3)	0 (−0.1 to 0)	10 098 (1328 to 18 869)	−36 148 (−52 188 to −20 109)	2.5 (1.0 to 4.1)	0.4 (−0.1 to 0.8)	71 673 (27 338 to 116 008)	74 525 (28 524 to 120 526)
Staff not wearing surgical masks or N95 respirators	257.4 (245.0 to 269.8)	541.6 (482.0 to 601.2)	7.9 (2.2 to 3.6)	0 (−0.1 to 0.2)	11.5 (4.8 to 18.2)		0.2 (0 to 0.4)	0.1 (0 to 0.1)	15 833 (6475 to 25 191)	−26 945 (−45 089 to −8801)	2.7 (1.2 to 4.2)	0 (−0.4 to 0.5)	77 109 (33 471 to 120 747)	79 950 (34 685 to 125 215)
35% of Staff who were infected get tested	163.6 (155.5 to 171.8)	315.1 (259.5 to 370.7)	3.3 (−1.4 to 8.0)	−0.1 (−0.2 to 0.1)	4.1 (−1.7 to 10.0)		0 (−0.2 to 0.1)	0 (−0.1 to 0)	5960 (−4509 to 16 430)	−28 624 (−46 312 to −10 937)	2.3 (0.7 to 3.8)	0.4 (0 to 0.8)	57 516 (12 153 to 102 879)	59 483 (12 403 to −106 563)
65% of Staff who were infected get tested	301.3 (289.6 to 313.0)	607.7 (548.6 to 666.8)	5.8 (1.5 to 10.1)	0 (−0.1 to 0.2)	8.1 (2.8 to 13.4)		0.1 (−0.1 to 0.3)	0 (−0.1 to 0)	13 159 (4006 to 22 311)	−43 688 (−59 979 to −27 396)	3.6 (2.0 to 5.1)	0.2 (−0.2 to 0.6)	95 077 (51 049 to 139 105)	98 967 (53 276 to 144 658)

Abbreviation: CMS, Centers for Medicare & Medicaid Services.

^a^ Data are presented as mean (95% CI).

^b^ Based on allowing 75% of staff who were mildly ill testing positive for SARS-CoV-2 to work while wearing N95 respirators compared with furloughing all staff who tested positive.

^c^ All resident outcomes were averted counts per year for a 100-bed nursing home and equivalent to a rate per 100 person-years.

^d^ Negative values are cost savings accrued when allowing 75% of staff who were mildly ill to work compared with furloughing all staff who tested positive for SARS-CoV-2.

Specifically, allowing 25% to 75% of staff who were mildly ill to work (staff who were moderately or severely ill were furloughed) alleviated understaffing. Compared with a 100% furlough, allowing 25% of staff who were mildly ill to work was associated with 6.1 (95% CI, 0.6-11.6) additional resident COVID-19 cases and no additional COVID-19–related hospitalizations or deaths. Conversely, additional working days allowed 120.2 (95% CI, 58.7-181.6) additional completed tasks, resulting in 2.1 (95% CI, 0.5-3.7) fewer non–COVID-19–related hospitalizations and 0.1 (95% CI, −0.4 to 0.5) fewer non–COVID-19–related deaths, decreasing overall costs by $54 270 to $72 490 (varying by perspective). When 75% of staff who were mildly ill worked while masked, the tradeoffs further favored preventing harm. There was an annual mean 240.0 fewer furlough days, 475.9 additional tasks completed of 649.5 missed tasks (73%), 3.5 fewer non–COVID-19–related hospitalizations, and 0.4 fewer non–COVID-19–related deaths compared with a 100% staff furlough (Table 2). Furthermore, this scenario was associated with 5 additional staff and 5 additional resident COVID-19 cases, but there was no association with additional COVID-19–related hospitalizations. This saved an annual total mean $85 470 (95% CI, $41 210-$129 730) from the CMS perspective and $134 450 (95% CI, $86 370-$182 540) from the societal perspective compared with a 100% staff furlough. Non–COVID-19–related costs outweighed COVID-19 costs across all perspectives when allowing 25% to 75% of staff who were mildly ill to work, ranging from $1 154 290 in non–COVID-19–related costs compared with $281 770 in COVID-19–related costs (4.1 times higher) to $1 134 700 in non–COVID-19–related costs compared with $130 100 in COVID-19–related costs (8.7 times higher).

### Increasing Vaccination Coverage

Doubling annual vaccination coverage among residents (to 76%) and staff (to 46%) in all scenarios was associated with approximately 16 to 19 fewer staff COVID-19 cases and 21 to 23 fewer resident COVID-19 cases annually, reducing COVID-19–related costs by $29 400 to $61 850 (varying by perspective). Reduced staff cases decreased furlough days by a mean 48.6 (95% CI, 34.7-62.5), missed tasks by a mean 116.1 (95% CI, 52.9-179.4), annual non–COVID-19–related hospitalizations by a mean 0.7 (95% CI, −0.9 to 2.2), and non–COVID-19–related costs from the CMS perspective by $17 670 (95% CI, −$26 210 to $61 550). Additionally, allowing 25% to 75% of staff who were mildly ill to work decreased missed tasks associated with COVID-19 furloughs by 149.0 to 416.5. Allowing 75% of staff who were mildly ill to work while masked resulted in fewer missed tasks, fewer non–COVID-19–related hospitalizations, and fewer non–COVID-19–related costs compared with a 100% staff furlough (Table 2). Increased vaccination reduced both COVID-19–related and furlough-related non–COVID-19 hospitalizations and deaths.

### Increasing Virus Transmissibility and Community Infection Risk

When evaluating whether increased SARS-CoV-2 transmissibility and community infection risk may change the estimated impact of allowing staff who were mildly ill to work, we found that increasing the transmission probability to 0.1 increased staff cases by 123.7 annually (22.5% community onset) and annual furlough days to 665 when 0% of staff who were mildly ill worked and to 167 annual furlough days when 75% of staff worked mildly ill (eFigure 3 in Supplement 1). Even with more staff and resident COVID-19 cases, allowing 75% of staff who were mildly ill to work while masked resulted in 984 additional completed tasks with significantly fewer non–COVID-19–related outcomes and no association with resident additional COVID-19 outcomes, reducing total costs (Table 2). Overall, alleviating non–COVID-19–related harms with fewer staffing shortages when 75% of staff who were mildly ill worked resulted in more cost savings despite increased COVID-19 cases when increasing viral transmissibility (Table 2).

Increasing the community infection risk 1.3 times the baseline values increased staff COVID-19 cases by 9 and resident cases by 3 annually. However, allowing staff who were mildly ill to work while masked was associated with few additional resident cases and COVID-19–related hospitalizations while also being associated with substantially fewer non–COVID-19–related harms from missed tasks, thus saving costs compared with a 100% staff furlough (Table 2).

### Increasing the Severity of COVID-19

Increasing COVID-19 illness severity (increasing the probability of hospitalization by 3 times) also did not change the direction of tradeoffs, with reduced furloughs, missed tasks, and non–COVID-19–related harms from allowing staff who were mildly ill to work still outweighing COVID-19 outcomes. For example, when allowing 75% of staff who were mildly ill to work, costs saved from averted non–COVID-19–related harms ($38 395) outweighed increased costs associated with resident COVID-19 cases ($13 121) from the CMS perspective (Table 2), saving $25 280 annually. Overall, societal cost savings from not furloughing staff were less than the current severity but still saved a mean $71 550 ($95% CI, $24 590-$118 510) per year.

### Staff Adherence to Masking While Working Mildly Ill and Percentage of Staff Being Tested

When accounting for the possibility that staff working with mild COVID-19 did not adhere to masking, the findings were robust when staff wore surgical masks instead of N95 respirators (Table 2). When staff who were mildly ill did not wear masks or N95 respirators, there were significantly more staff and resident COVID-19 cases but not hospitalizations, as well as additional CMS costs ($15 833). These costs were still outweighed by the cost savings from completed tasks that were associated with the prevention of non–COVID-19–related harms ($77 109). Similarly, the results were robust to changes in the percentage of staff who were infectious getting tested and to increased vaccination coverage, nursing home transmission, and importation of COVID-19 from the community (Table 2).

## Discussion

Pervasive staffing shortages currently limit the provision of basic care needs in US nursing homes.^42,43^ Our simulation experiments in this modeling study resulted in an annual 22.1% deficiency in care tasks at current staffing levels. Our simulations suggest that a mandatory staff furlough for COVID-19 illness may have exacerbated current understaffing and was associated with additional non–COVID-19–related hospitalizations and potentially an additional death per year (0.7) among residents in a 100-bed nursing home associated with missed care tasks. In contrast, if staff with mild COVID-19 illness worked while masked, 73% of those missed care tasks could be completed with the tradeoff of a small number of additional COVID-19 illnesses with minimal COVID-19–related hospitalizations for either staff or residents. Thus, under current staffing conditions, furloughing staff who were mildly ill with COVID-19 was associated with non–COVID-19–related harms that outweighed COVID-19–related harms among nursing home residents.

Changing current regulation to allow staff with mild COVID-19 to work with an N95 respirator also may be cost saving for both the CMS and society. For each 100-bed nursing home, the CMS could save $85 470 from harms and hospitalizations. With 15 000 US nursing homes, such a change in regulation could translate to savings well over $1 million for the CMS while alleviating substantial harm.

The intent of regulations to furlough all staff with COVID-19 earlier in the pandemic was to protect staff and residents when COVID-19 frequency, severity, and sequelae were much greater. Since then, vaccination and prior illness have substantially reduced severe outcomes. This in turn may have tipped the balance between the benefits of mandatory furlough and unintended consequences on resident care, as well as the balance between staff furlough and burnout of remaining staff.^42,44,45^

Our simulation experiments may be conservative. We assumed staff were unlikely to disclose illness and would work with hidden symptoms or presume mild symptoms were from noninfectious ailments. Thus, in our experiments, only half of staff infected with SARS-CoV-2 reported or had sufficiently overt symptoms to trigger testing. The rest of the staff worked while contagious, thus undermining the current mandated furlough policy. Reasons for underreporting and working ill include a need for workers to protect limited paid sick days. Substituting a policy that allows staff who are mildly ill to work could renew and compel efforts to train staff on masking; frequent hand disinfection; and distancing, when able, from coworkers and residents. Such a policy may also reduce staff turnover.^46,47,48^

All of this is not to say that nursing home employees should be encouraged to work while sick with a contagious pathogen. Rather, our work underscored the reality that the development of appropriate nursing home staffing policies involves tradeoffs (eg, chronic understaffing means that reducing available staff time can have consequences). Models can help with assessing tradeoffs such as whether worsening understaffing by mandated furloughs may lead to more or less harm than allowing staff to work while contagious. We found that effective vaccines (representing current annual uptake), acculturation to masks (access, expectations, and the ability to wear them), and SARS-CoV-2 strain adaptations may now allow a clear answer of worsening harm from COVID-19 furloughs. This finding was unchanged when increasing virus transmissibility or severity, with non–COVID-19–related costs outweighing COVID-19–related harms and costs from all perspectives. This is because even a greater increase of COVID-19–related hospitalizations in a population that was relatively immune did not approach the severity experienced when SARS-CoV-2 entered a fully susceptible population in 2020. Other models can similarly assess such tradeoffs for other pathogens, as these results may not be generalizable beyond COVID-19.

This study constitutes an important example of how infection-control decision-making should account for broader and downstream impacts throughout a system and how systems approaches can elucidate these impacts.^49^ The ultimate solution would be to substantially increase nursing home staff. This would not only address the issue of missed tasks even absent infectious disease threats, but would allow greater leeway for furlough strategies to control pathogen spread. New federal quality standards require 3 nursing hours per resident day (0.55 hours of RN care, 2.45 hours of nurse aide care).^50^ Another study has estimated that over 4 nursing hours are needed for quality care.^51^ However, most nursing homes are severely understaffed with wage levels unable to compete with some fast food opportunities.^42,43^ The pandemic further exacerbated pervasive staffing shortages, which have persisted.^8,42,52,53^ In 2021, 94% of nursing homes were understaffed; in 2024, 72% had fewer staff than before the pandemic. If nursing homes become sufficiently staffed to handle cross-coverage for furloughing staff while preventing burnout,^42,54^ then the tipping point may change.

### Limitations

Limitations of our study include that all models are simplifications of actual situations and cannot account for every scenario and outcome. We made multiple conservative assumptions. We assumed that a non–COVID-19–related outcome was associated with a specific missed task; however, outcomes may be associated with and exacerbated by multiple missed tasks. Furthermore, residents may experience multiple negative outcomes; however, we assumed residents incurred only the first. We assumed that staff performed tasks at the same efficiency regardless of staffing; in reality, staff may initially complete tasks more quickly if short-staffed but may ultimately burn out. Additionally, we did not represent prioritizing residents with a higher risk level or residents not attended to recently. We also assumed specialty care staff would not perform basic care; however, they may if greatly understaffed. Lastly, residents only accrued costs and health effects for hospitalizations and deaths. However, missed tasks may be associated with other detrimental effects to quality of life and costs.

## Conclusions

This modeling study found that mandatory furlough of nursing home staff with mild COVID-19 illness was associated with more resident harm from missed care tasks than with harm from increased COVID-19 transmission. In the current climate of extreme nursing home understaffing, allowing those with mild COVID-19 illness to work may prevent more harm from staffing shortages and missed care tasks, ultimately saving substantial costs.

## Notes

Supplement 1.
eMethods
**eFigure 1.** Agent-Based Model Overview: NH Structure and Agent Mixing and Movement **eFigure 2.** Agent-Based Model Overview: (A) SARS-CoV-2 Infection Pathway for Unvaccinated and Vaccinated NH Residents; and (B) SARS-CoV-2 Infection Pathway for Unvaccinated and Vaccinated NH Staff **eTable 1.** Nursing Home Mixing Parameters **eTable 2.** Model Input Parameters, Values, and Sources for Nursing Home Characteristics **eTable 3.** Model Input Parameters, Values, and Sources for Virus and Transmission Characteristics **eTable 4.** Model Input Parameters, Values, and Sources for COVID-19 Policy and Intervention Characteristics **eTable 5.** Model Input Parameters, Values, and Sources for Nursing Home Staff Characteristics and COVID-19 Health and Clinical Outcomes **eTable 6.** Model Input Parameters, Values, and Sources for Nursing Home Resident Health and Clinical Outcomes **eFigure 3.** Impact of Furloughing Staff Testing Positive for COVID-19 When Assuming Greater Virus Transmissibility (Probability of Transmission 0.1 per Contact) on the Average Number of (A) Missed Resident Care Tasks, (B) Resident Hospitalizations, (C) Resident Deaths, (D) Costs Incurred From the CMS Perspective, and (E) Costs Incurred From the Societal Perspective
eReferences

Supplement 2.
Data Sharing Statement
