# Supplementary material for: Modeling Nursing Home Harms From COVID-19 Staff Furlough Policies
Source: JAMA Netw Open. 2024 Aug 19;7(8):e2429613. doi: 10.1001/jamanetworkopen.2024.29613 (PMC11333984; doi:10.1001/jamanetworkopen.2024.29613)
Supplement: Supplement 1. — eMethods eFigure 1. Agent-Based Model Overview: NH Structure and Agent Mixing and Movement eFigure 2. Agent-Based Model Overview: (A) SARS-CoV-2 Infection Pathway for Unvaccinated and Vaccinated NH Residents; and (B) SARS-CoV-2 Infection Pathway for Unvaccinated and Vaccinated NH Staff eTable 1. Nursing Home Mixing Parameters eTable 2. Model Input Parameters, Values, and Sources for Nursing Home Characteristics eTable 3. Model Input Parameters, Values, and Sources for Virus and Transmission Characteristics eTable 4. Model Input Parameters, Values, and Sources for COVID-19 Policy and Intervention Characteristics eTable 5. Model Input Parameters, Values, and Sources for Nursing Home Staff Characteristics and COVID-19 Health and Clinical Outcomes eTable 6. Model Input Parameters, Values, and Sources for Nursing Home Resident Health and Clinical Outcomes eFigure 3. Impact of Furloughing Staff Testing Positive for COVID-19 When Assuming Greater Virus Transmissibility (Probability of Transmission 0.1 per Contact) on the Average Number of (A) Missed Resident Care Tasks, (B) Resident Hospitalizations, (C) Resident Deaths, (D) Costs Incurred From the CMS Perspective, and (E) Costs Incurred From the Societal Perspective eReferences [file jamanetwopen-e2429613-s001.pdf]

Supplemental Online Content

Bartsch SM, Weatherwax C, Leff B, et al. Modeling nursing home harms from COVID-19 staff furlough policies. *JAMA Netw Open*. 2024;7(8):e2429613.
doi:10.1001/jamanetworkopen.2024.29613

eMethods

eFigure 1. Agent-Based Model Overview: NH Structure and Agent Mixing and Movement

eFigure 2. Agent-Based Model Overview: (A) SARS-CoV-2 Infection Pathway for Unvaccinated and Vaccinated NH Residents; and (B) SARS-CoV-2 Infection Pathway for Unvaccinated and Vaccinated NH Staff

eTable 1. Nursing Home Mixing Parameters

eTable 2. Model Input Parameters, Values, and Sources for Nursing Home Characteristics

eTable 3. Model Input Parameters, Values, and Sources for Virus and Transmission Characteristics

eTable 4. Model Input Parameters, Values, and Sources for COVID-19 Policy and Intervention Characteristics

eTable 5. Model Input Parameters, Values, and Sources for Nursing Home Staff Characteristics and COVID-19 Health and Clinical Outcomes

eTable 6. Model Input Parameters, Values, and Sources for Nursing Home Resident Health and Clinical Outcomes

eFigure 3. Impact of Furloughing Staff Testing Positive for COVID-19 When Assuming Greater Virus Transmissibility (Probability of Transmission 0.1 per Contact) on the Average Number of (A) Missed Resident Care Tasks, (B) Resident Hospitalizations, (C) Resident Deaths, (D) Costs Incurred From the CMS Perspective, and (E) Costs Incurred From the Societal Perspective

eReferences

This supplemental material has been provided by the authors to give readers additional information about their work.

© 2024 Bartsch SM et al. *JAMA Network Open*.

eMethods

SARS-CoV-2 Transmission

eFigure 1 shows the previously described[1,2] mutually exclusive SARS-CoV-2 states that each agent could be in and how agents moved through them. At the start of the simulation, all agents are uninfected. Staff had a daily probability of infection in the community and could subsequently introduce SARS-CoV-2 into the nursing home (NH). Each day, agents interacted with each other, and an infectious person could transmit SARS-CoV-2 to a susceptible person. If a susceptible agent came into effective contact (i.e., interacts and transmits SARS-CoV-2) with an infectious agent, they became exposed and ultimately infected. The following example equation governed if a susceptible resident became infected:

$$1-((1-\text{Daily Contact Probability Resident To Routine Staff} * \text{Transmission Probability})^{NumberInfectedRoutineStaff} * (1-\text{Daily Contact Probability Resident To Specialty Staff} * \text{Transmission Probability})^{NumberInfectedSpecialtyStaff} * (1-\text{Daily Contact Probability Resident To Social Residents} * \text{Transmission Probability})^{NumberInfected\ SocialResidents} * (1-\text{Daily Contact Probability Resident To Roommate} * \text{Transmission Probability})^{NumberInfectedRoommates})$$

Daily contact probabilities were defined as at least 15-minute contacts within 6 feet in which could potentiate the transmission of the virus. We calibrated the transmission probability, and assumed it was the same between any two individuals per contact (i.e., did not vary based on type or intensity of actions). Overall, an agent's probability of becoming infected depended on the frequency of interactions. Further, staff-to-staff interactions were attenuated to account for other ongoing precautions (e.g., hand hygiene, physical distancing). Each infected individual became infectious prior to disease onset. After recovering, agents had natural immunity against infection (waned over time; eTable 4) and against hospitalization (long-lasting[3]).

Costs and Economic Outcomes

Each person accrued relevant costs and health effects as they moved through the model. If a resident incurred more than one negative health outcome due to missed tasks, they incurred the first outcome based on the time to harm. The model generates health effects and costs from the Centers for Medicare and Medicaid Services (CMS), all third-party payers combined, and societal perspectives. The CMS perspective included direct medical costs for residents (e.g., doctor visits, laboratory tests, antiviral medications, hospitalization). The third-party payer perspective included direct medical costs for residents and staff, and the societal perspective included direct and indirect (i.e., productivity losses due to presenteeism, absenteeism) costs. Hourly wage served as a proxy for productivity losses. Absenteeism resulted in productivity losses for the duration of isolation or hospitalization, while presenteeism (wages attenuated by utility weights) resulted in productivity losses for staff working while ill. All COVID-19 cases accrued productivity losses, as everyone is assumed to contribute to society. Health effects were measured in quality-adjusted life-years (QALYs) lost. Each agent lost QALYs based on the age-dependent healthy QALY value and severity-specific utility weights for his/her infection duration and hospitalization duration for experienced harm. Death resulted in the loss of the net present value of QALYs for the remainder of an individual's lifetime.[4] All costs were reported in 2024 values, converting all past and future costs using a 3% annual rate.

Data Sources

eTables 1-6 show our model input parameters, values, and sources, with each table divided by type of parameter (e.g., probabilities, costs, durations). All inputs came from the scientific literature and nationally representative data sources, when available data allowed, and we supplemented with expert opinion, as described below. Additionally, all inputs were age-specific when available.

First, we identified data sources to populate the NH structure (e.g., number of rooms, number of agents) and agent mixing/moving. eTable1 shows the parameters that govern how NH residents and staff interact with each other, which we derived from empirical data on daily contacts observed in Orange County, CA NHs.[1,2] eTable2 shows parameters for the characteristics of the NH, including the number of staff and residents, and the types of NH staff. For example, the number of residents, resident length-of-stay, and the proportion of post-acute care residents came from national databases (e.g., CMS Minimum Data Set), while the number of bed-bound resident rooms (i.e., non-mixing resident rooms) came from the scientific literature. We obtained summary estimates for the number and types of staff from a survey of ten Southern California NHs (which provide skilled nursing care) based on staffing records and direct observations (10 NH Survey).

© 2024 Bartsch SM et al. *JAMA Network Open*.

Next, we populated the SARS-CoV-2 virus and transmission parameters as well as the COVID-19 health, clinical, and economic related parameters. Specifically, eTable3 shows the virus and transmission characteristics, while eTable 4 shows parameters related to various COVID-19 interventions (e.g., testing, vaccination), and eTable5 and eTable6 show parameters related to COVID-19 health and clinical outcomes for staff and residents, respectively. These parameter values came from the scientific literature and national databases (e.g., Healthcare Cost Utilization Project [HCUP], RedBook) and are specific for the Omicron variant. We calibrated the probability of transmission per contact with an infectious agent (described below). We estimated the daily risk of SARS-CoV-2 infection from the community assuming an early January winter peak similar to that of winter 2023-2024.

We then found data sources to parameterize model inputs, shown in eTable5, pertaining to how staff perform the various resident care tasks, including the time it takes to do each task, and the time they have available to perform these tasks, and how staff prioritize these resident care tasks. In order to obtain values for these parameters, we conducted a literature search using MEDLINE/PubMed and Google Scholar with key terms including but not limited to "nursing homes", "staff time", "staff productivity", "staff efficiency", "missed care tasks", "missed wound care", "lack turning", "missed medications", "missed bathing", "missed hygiene care", "toileting assistance", "exercising/walking", "missed feeding", for studies (restricted to the English language) published between 2000 and 2024 assessing NH staffing, NH staff time, and missed resident care tasks. We reviewed identified studies for relevance and extracted data (e.g., year of data collection, location, population, sample size, counts/percentages/rates of key outcomes) from each. We referenced studies conducted in the United States (US) and systematic reviews; when not available, we supplemented with data from other countries (e.g., Italy, Finland). We obtained summary estimates for the time needed to complete daily tasks from the 10 NH Survey.

Lastly, we identified data sources for parameters related to the harm residents could experience if NH staff did not complete specific care tasks (eTable6). In order to obtain values of these parameters, we conducted another literature search. We searched MEDLINE/PubMed and Google Scholar (again limiting to the English language and those published between 2000 and 2024) using key terms including but not limited to "nursing homes", "resident outcomes", "resident missed care", "missed task consequences", "dehydration", "infection", "pressure sores", "risk hospitalization", "avoidable hospitalizations", "time to hospitalization", "exercise/walking", "falls", and "broken bones", for studies assessing harm outcomes from missed tasks for NH residents, the probability/risk of hospitalization, and the time/days to harm for each harm related outcome. In the absence of finding studies reporting the number of days tasks would need to be missed before residents experience harm and the probability of hospitalization for each type of harm, estimates for these parameters came from discussions with geriatricians (BL, MRW) who have substantial expertise and clinical experience in NHs. BL directs the Center for Transformative Geriatric Research and Professor of Medicine at the Johns Hopkins University School of Medicine. He has over 30 years of geriatric clinical experience, including 10 years as the attending physician on a 60-bed NH unit. MRW chairs the Public Policy Committee at the California Association of Long Term Care Medicine, and was previously a board member for the American Geriatrics Society's Foundation for Health in Aging. He has over 30 years of experience in older adult medical care, including serving as NH medical director for many facilities. The experts synthesized available data on the harms stemming from missed activities and their own clinical experience.[5-14] Days-to-harm reflected clinically relevant estimates for the number of days of missed tasks after which a NH resident would experience harm (e.g., missed feeding/hydrating tasks for 1-3 days can lead to dehydration), accounting for a range of underlying health conditions, and are longer than the recommended times between resident care tasks.[15] Similarly, the probability of hospitalization accounts for a range of underlying health conditions. For each type of harm, the hospital length-of-stay and probability of mortality came from national databases (i.e., CMS Minimum Data Set and ResDAC), while the cost per hospital bed day came from HCUP. We conducted a search of the Tufts CEA Registry to identify studies reporting utility weights for each of the specific harm outcomes.

Model Calibration and Validation

We systematically conducted validation of the model at multiple levels. The first level of validation was at the staff behaviors and actions level. This entailed determining how well various model generated staffing and task parameters could approximate observed NH staffing and missed resident care tasks. For example, we determined how well model-generated staff hours available per resident based on the inputs obtained from the 10 NH Survey could match published NH staffing levels. Our model inputs for the number of full-time and part-time staff and duration of each shift correspond to 2.66 staff hours available per resident (for CNAs, RNs, and LPNs), including night shifts. This is similar to the 2.57 total identified for RNs and CNAs in a quality improvement study of 13,339 U.S. NHs[16], and the mean 2.48 total hours identified in a study of 85 U.S. NHs.[17] When accounting only for the

© 2024 Bartsch SM et al. *JAMA Network Open*.

represented day and evening shifts and 80% of staff time spent on care tasks, this equated to 1.74 hours per resident available. As another example, we compared the model generated number of missed care tasks to the estimated missed care tasks in the scientific literature. This included validating the observed times to complete resident care tasks from the 10 NH survey, which were similar to the ranges used in a discrete event staffing simulation model.[18] In the absence of SARS-CoV-2, our model generated an average of 93.7 missed tasks per day (22.1% of all tasks) due to understaffing, corresponding to 3.0 missed tasks per shift. This is within the range identified by a study interviewing NH staff of 0-9 (average 1.22, SD 1.73) missed tasks.[19] However, this study did not include all of the resident care tasks that we considered, such as mobility care/exercising/walking residents. Additionally, our simulated 22.1% of tasks not being completed is comparable with studies in other countries. A study of missed tasks in Dutch NHs found that 36% of total care tasks were missed (14.5% of which were routine care) and another in an Italian NH found that 17.1% of care tasks were missed.[20,21] Again, these studies did not include tasks such as exercise/walking residents and thus may underestimate the percent of missed tasks compared to our model generated output which included these tasks.

The second level of validation was at the resident behaviors and actions level. For example, this entailed determining how well the model generated number of hospitalizations due to missed care tasks matched published estimates on potentially avoidable hospitalizations among NH residents. Our model generated an average of 5.2% of residents hospitalized due to missed care tasks per year. This is similar to a study that analyzed national U.S. data from 2006-2008 and found that 7,595 hospitalizations that should have been avoidable with high quality nursing care (which includes infection control, falls prevention, skin and wound care, medication management, and meeting residents' dietary and nutritional needs) among 62,745 long-stay NH residents over two years, which translates to 6.1% of residents hospitalized annually.[22] Our model also generated an average of 3.2 nonCOVID-related hospitalizations per month for a 100-bed NH, which is within the range of potentially avoidable hospitalizations per month for a 100-bed NH, 2.25 to 4.5, found in a study of 85 US NHs.[17]

The third level of validation was at the SARS-CoV-2 transmission level. We calibrated the transmission probability by varying its value across a plausible range so that approximately 70% of staff would be infected with SARS-CoV-2 over the course of a year, with about 47% of staff experiencing sufficiently overt symptoms to warrant testing. This corresponded to a transmission probability of 3% per contact with an infectious agent. Validation of SARS-CoV-2 transmission involved determining how well the simulated number of staff and resident cases and hospitalizations could approximate historic cases and hospitalizations. For example, the model generated an average of 21.5 staff infections and 14.4 staff symptomatic cases per 1,000 resident weeks. These model-generated estimates are consistent with a 2-2.5 underreporting factor of the 12.4 test-confirmed staff cases per 1,000 resident weeks during the Winter peak in 2023 and 10.9 in January 2024 reported by the Centers for Disease Control and Prevention (CDC).[23] A 3% transmission probability also resulted in approximately 11% of residents infected over one year (if no staff worked while sick), which corresponds to an average of 1.6 resident cases per week in the 100-bed NH. This is similar to the 0.61 to 1.33 per 100 beds per week in U.S. NHs from October 2023 to February 2024.[24] Further, our model also generated 0.012 resident COVID-19 hospitalizations per week, which is similar to the cumulative average 0.058 per week from October 2023 to February 2024.[24]

© 2024 Bartsch SM et al. *JAMA Network Open*.

eFigure 1. Agent-Based Model Overview: NH Structure and Agent Mixing and Movement

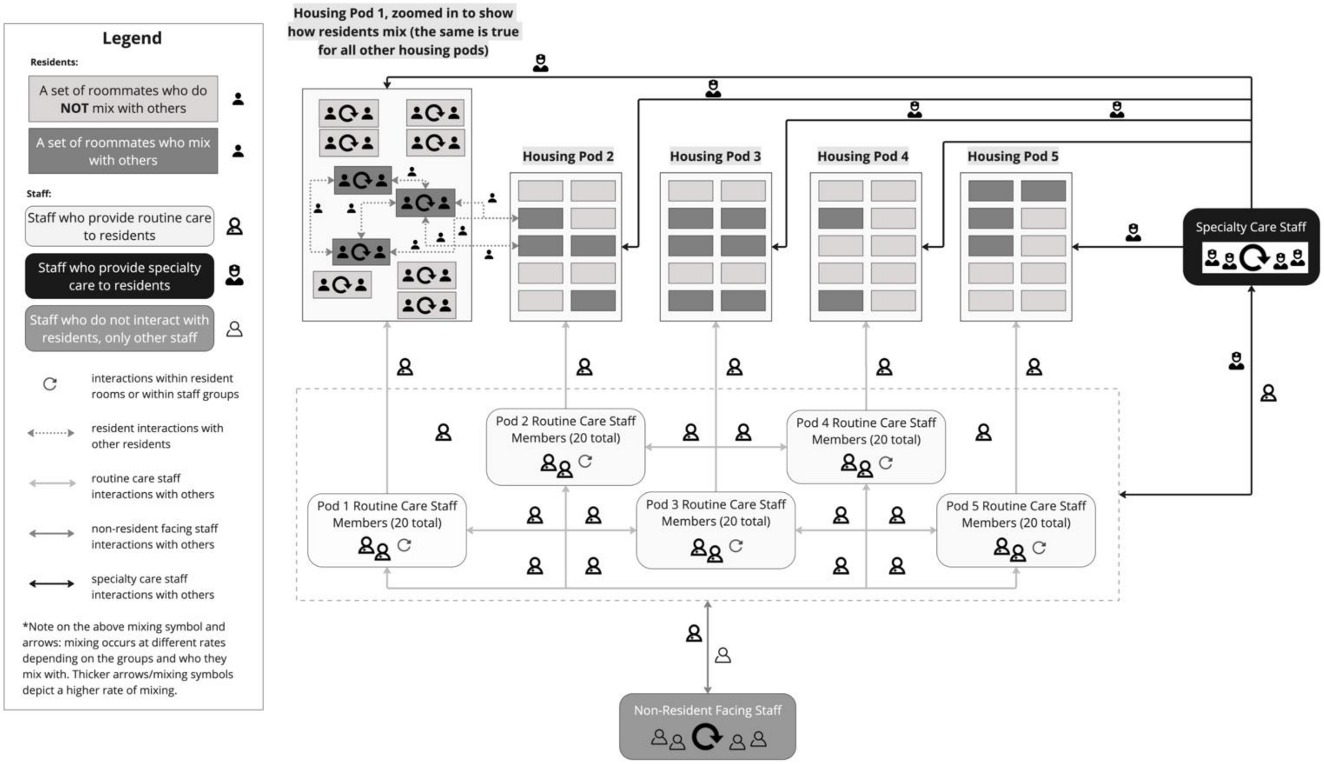

© 2024 Bartsch SM et al. *JAMA Network Open*.

eFigure 2. Agent-Based Model Overview: (A) SARS-CoV-2 Infection Pathway for Unvaccinated and Vaccinated NH Residents; and (B) SARS-CoV-2 Infection Pathway for Unvaccinated and Vaccinated NH Staff

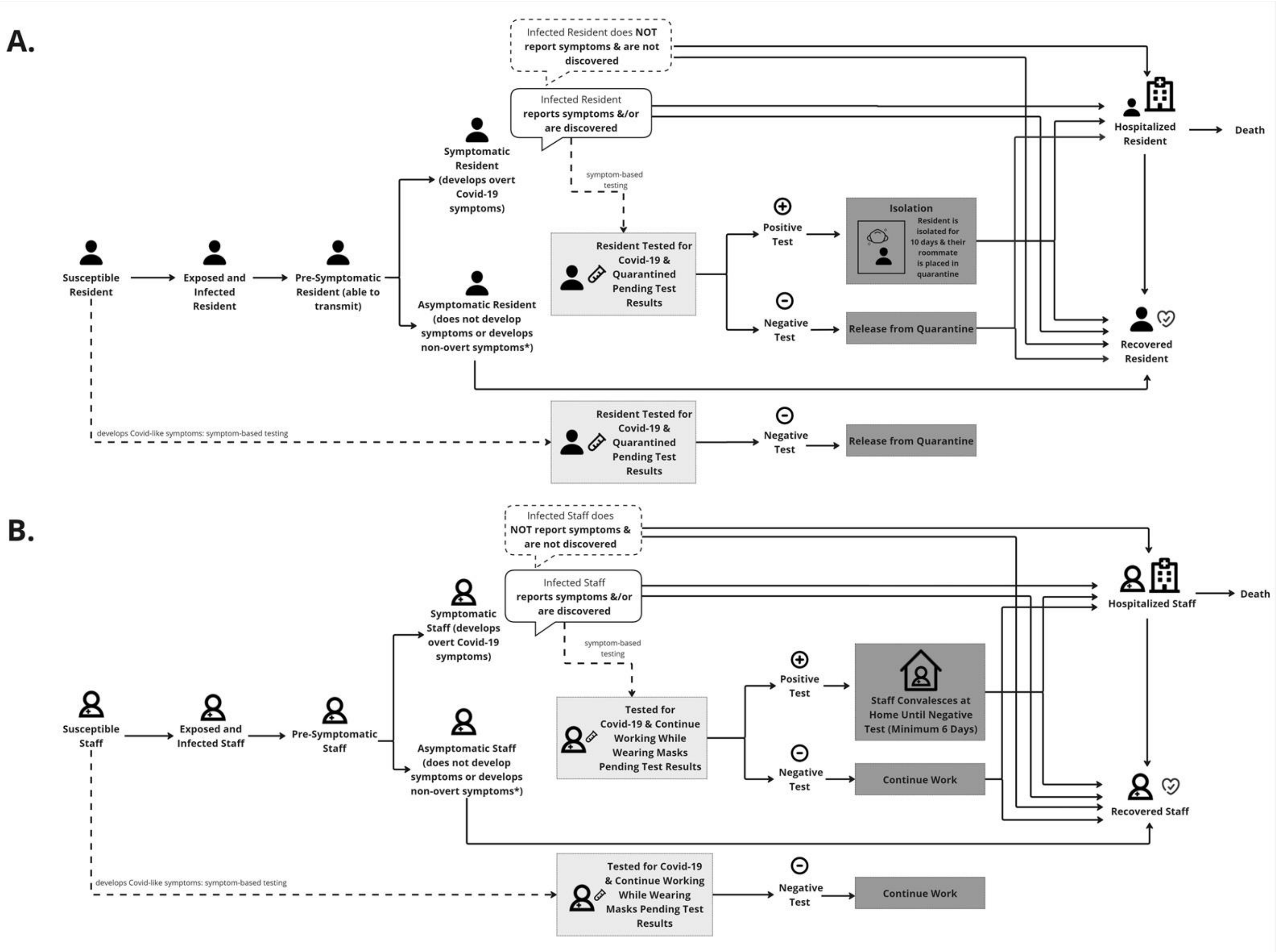

© 2024 Bartsch SM et al. *JAMA Network Open*.

eTable 1. Nursing Home Mixing Parameters

Nursing Home Population Mixing Parameters			
**Contact from**	**Contact with**	**Daily contact probability between groups**	**Average exposures per day**
Resident	Resident's roommate	1[a]	88
Resident[b]	Specialty staff	0.072	7.2
Social residents	Social residents (excluding roommate)	0.1	6
Resident	Routine staff	0.385	7.7
Resident	Non-resident facing staff	0	0
Routine staff	Other routine staff	0.051	4.3
Routine staff	Specialty staff	0.023	0.4
Routine staff	Non-resident facing staff	0.054	1.7
Specialty care staff	Other specialty care staff	0.26	4.7
Specialty care staff	Non-resident facing staff	0	0
Non-resident facing staff	Other non-resident facing staff	0.24	7.1

[a] Assumes roommates will have a 100% probability of contact in a given day

[b] Value for all residents; however, specialty care staff only interact with residents with a length of stay <100 days (post-acute care residents)

Note: The daily contact probabilities between individuals derived from empirical data on the number of daily contacts for residents and staff from surveyed and observed interactions in Orange County, CA NHs. We counted the number of effective contacts, defined as 15 minute or longer interactions within 6 feet. Using detailed shift-based data, we calculated daily contact probabilities by dividing the weekly number of contacts within and across residents and staff types by the number of staff working that week and distributing across 7 days.

© 2024 Bartsch SM et al. *JAMA Network Open*.

eTable 2. Model Input Parameters, Values, and Sources for Nursing Home Characteristics

**Nursing Home Characteristics**				
**Parameter**	**Distribution Type**	**Mean/ Median**	**Range/Standard Deviation**	**Source**
Number of residents[a]	Point Estimate	100	–	[25]
Number of routine care staff (e.g., certified nursing assistants, licensed vocational and registered nurses, environmental services workers)	Point Estimate	100	–	10 NH Survey
CNAs	Point Estimate	40 (FTE: 35)	–	10 NH Survey
RNs/LPNs	Point Estimate	25 (FTE: 18)	–	10 NH Survey
CNAs (day shift only)	Point Estimate	35 (FTE: 29)		10 NH Survey
RNs/LPNs (day shift only)	Point Estimate	20 (FTE: 14)		10 NH Survey
Number of specialty care staff (e.g., physical, occupational, and speech therapists)	Point Estimate	20	–	10 NH Survey
Number of non-resident facing staff (e.g., medical records, office and administrative support)	Point Estimate	30	–	10 NH Survey
Resident length of stay	Gamma	69.7	250.2	[26]
Number of non-mixing (bed-bound) resident rooms	Point Estimate	10	–	[27]
Proportion of post-acute care residents (length-of-stay <100 days)		0.82		[26]
Proportion of residents and staff who have had COVID-19 in the last 6 months and have some level of immunity at the start of the model	Point Estimate	0.2	–	Expert Opinion
Number of staff needed to care for residents with harm-related outcomes (if below this value, residents will be hospitalized due to insufficient staffing)	Point Estimate	50	–	Calculated[b]

[a] Each day, new individuals enter the NH such that the number of staff and residents remains constant (e.g., new resident admissions equals resident deaths and bed turnovers). New residents and staff entering the model have a probability of having recovered from COVID-19 within the last 6 months and having pre-existing immunity.
[b] Calculated by adding up shifts (which equaled about 4 staff hrs/resident/day) and using a threshold of minimum 2 hrs/resident/day

© 2024 Bartsch SM et al. *JAMA Network Open*.

eTable 3. Model Input Parameters, Values, and Sources for Virus and Transmission Characteristics

**Virus and Transmission Characteristics**				
**Parameter**	**Distribution Type**	**Mean/ Median**	**Range/Standard Deviation**	**Source**
Incubation period (days)	Gamma	3.5	2.4	[28-31]
Days can transmit prior to disease onset	Point Estimate	2	–	[32]
Infectious period (days)	Beta Pert	5	3 – 9	[33]
Probability of transmission given effective contact	Point Estimate	0.03	–	Calibrated
Staff to staff reduction in mixing/transmission for other precautions (e.g., hand hygiene, physical distancing)	Point Estimate	0.70	-	Calibrated

© 2024 Bartsch SM et al. *JAMA Network Open*.

eTable 4. Model Input Parameters, Values, and Sources for COVID-19 Policy and Intervention Characteristics

Parameter	Distribution Type	Mean/ Median	Range/Standard Deviation	Source
**COVID-19 Policy and Intervention Characteristics**				
**Probabilities**				
Vaccination coverage after vaccination campaign with current annual vaccine[a] (coverage is 0% a simulation start)				
Staff	Point Estimate	0.229	–	[34]
Residents	Point Estimate	0.381	–	[34]
Vaccine efficacy against infection (0-3 months)	Point Estimate	0.65	–	Assumption[b]
Vaccine efficacy against infection (ending protection after 6 months)	Point Estimate	0.325	–	Assumption[c]
Vaccine efficacy against hospitalization		0.761	0.623 – 0.848	[35]
Natural immunity against infection, starting protection in months 0-3	Point Estimate	1	–	[36]
Natural Immunity against infection, ending protection after 6 months	Point Estimate	0.30	–	[37]
Natural immunity against hospitalization	Beta Pert	0.819	0.738 – 0.880	[37]
N95 respirator efficacy	Point Estimate	0.99	–	[38]
N95 mask compliance	Point Estimate	0.75	–	[39] and [d]
Antigen test sensitivity	Beta Pert	0.81	0.78 – 0.84	[40]
Antigen test specificity	Point Estimate	1.0	–	[40,41]
Infectious staff report symptoms and tested for COVID-19	Point Estimate	0.5	–	[42,43]
Infectious resident report/show symptoms and tested for COVID-19	Point Estimate	0.3	–	Expert Opinion
**Durations**				
Minimum furlough length (days) given positive SARS-CoV-2 test for staff	Point Estimate	7	–	[44]
Isolation length (days) given positive SARS-CoV-2 test for resident	Point Estimate	10	–	Assumption
NH bed held (days) during resident hospitalization	Point Estimate	10	–	Assumption
Vaccination campaign start date		Oct 1		Assumption
Vaccination campaign duration		60 days		Assumption
**Costs (2024 US$)**				
COVID-19 antigen test	Point Estimate	12	–	[45]
Personal protective equipment (per interaction)				
N95 respirator (each)	Uniform	–	0.76 – 1.42	[46]
Gloves (per set of two)	Uniform	–	0.12 – 0.37	[47,48]
Gowns (each)	Uniform	–	1.06 – 1.64	[47,49]
Goggles / Face Shield	Uniform	–	2.08 – 2.50	[47,50]

[a] Vaccination coverage of residents and staff reported through January 7, 2024
[b] Assumed similar to flu vaccine efficacy.
[c] Assumed 50% reduction.
[d] Accounts for unmasked mealtimes

© 2024 Bartsch SM et al. *JAMA Network Open*.

eTable 5. Model Input Parameters, Values, and Sources for Nursing Home Staff Characteristics and COVID-19 Health and Clinical Outcomes

Parameter	Distribution Type	Mean/ Median	Range/Standard Deviation	Source
**Nursing Home Staff Characteristics and COVID-19 Health and Clinical Outcomes**				
**Probabilities**				
Leave job, daily (turnover not due to COVID-19)	Point Estimate	0.00017	–	[51]
Seeking ambulatory care for COVID-19	Beta Pert	0.167	0.150 – 0.184	[52]
Getting Paxlovid or any other COVID medications if seek ambulatory care	Beta Pert	0.284	0.2556 – 0.3124	[53]
Hospitalization for COVID-19 (unvaccinated)	Triangular	0.025	0.0225 – 0.0275	Assumption,[54]
ICU admission for COVID-19, given hospitalization	Beta Pert	0.095	0.0855 – 0.1045	[55]
Mortality, COVID-19, given hospitalization	Beta Pert	0.023	0.0207 – 0.0253	[55]
**Durations/Times**				
Staff shift length (excluding breaks)	Point Estimate	7 hours	–	Assumption,[18]
Percent of time per shift spent on resident care tasks	Point Estimate	0.8	–	Expert Opinion
Time needed for tasks of daily living, per resident, per day				
Exercising/moving non-bed-bound residents	Point Estimate	35 minutes	–	10 NH Survey
Exercising/moving bed-bound residents	Point Estimate	28 minutes	–	10 NH Survey
Feeding/hydrating[a]	Point Estimate	36 mins	–	10 NH Survey
Giving medications[b]	Point Estimate	30 mins	–	10 NH Survey
Turning (bed-bound residents)[c]	Point Estimate	10 mins	–	10 NH Survey
Providing toileting assistance or other hygiene related tasks[d] such as wound care[e]	Point Estimate	38 mins	–	10 NH Survey
Percent of RN time spent on medication-related tasks	Point Estimate	0.7		Calibrated[f]
Task not getting done if not enough time				
Hygiene tasks	Point Estimate	0.2		Expert Opinion,[20,56,57]
Turning tasks	Point Estimate	0.2		Expert Opinion,[20,56,57]
Feeding tasks	Point Estimate	0.1		Expert Opinion,[20,56,57]
Exercise tasks	Point Estimate	0.5		Expert Opinion,[20,56,57]

© 2024 Bartsch SM et al. *JAMA Network Open*.

**Costs (2024 US$)**				
Daily wage, NH staff				
Routine care	Triangular	220.12	97.26 – 490.65	58
Specialty care	Triangular	369.66	247.57 – 518.65	58
Non-resident facing	Triangular	138.34	91.15 – 254.70	58
Ambulatory care visit	Triangular	133.66	94.81 – 188.56	59
Over the counter medications, per dose	Gamma	0.108	0.417	60
Paxlovid	Point Estimate	1,668	-	60
Hospitalization of staff for COVID-19	Gamma	22,155.04	296.13	61
**Utility Weights**				
Healthy QALY 18-64 years old	Point Estimate	0.92	–	62

[a] Observed feeding/hydration tasks on average last 12 min and occur 3x per day.
[b] Observed medication tasks on average last 15 min and occur 3x per day (includes assessment time). We adjusted this to 2x per day.
[c] Observed turning tasks on average last 5 min and occur 3x per day. We adjusted this to 2x in day.
[d] Assumed that bathing residents occurred three times per week.
[e] Observed toileting/hygiene tasks on average last 6 min and occur 3x per day, and wound care tasks on average last 10 mins and occur 2x per day.
[f] Calculated using the proportion of time needed for resident medication tasks compared to other tasks and number of RN hours available.

© 2024 Bartsch SM et al. *JAMA Network Open*.

eTable 6. Model Input Parameters, Values, and Sources for Nursing Home Resident Health and Clinical Outcomes

Parameter	Distribution Type	Mean/ Median	Range/Standard Deviation	Source
**Nursing Home Resident Health and Clinical Outcomes**				
**Probabilities**				
Resident characteristics:				
Bed-bound	Point Estimate	0.20	–	[27]
Residents requiring feeding assistance/monitoring, per day	Point Estimate	1	–	10 NH Survey,[63]
Residents requiring hygiene assistance (e.g., bathing), per day[a]	Point Estimate	0.74	–	10 NH Survey,[63]
Residents requiring turning, per day[b]	Point Estimate	0.5	–	10 NH Survey,[63]
Residents who need exercise/moving assistance, per day	Point Estimate	1	–	10 NH Survey,[63]
Residents taking essential medications, per day	Point Estimate	1	–	10 NH Survey,[22]
Other respiratory illnesses, daily	Point Estimate	0.0015	–	Calibrated[c]
Asymptomatic / non-overt SARS-CoV-2 infection[d]	Beta Pert	0.324	0.253 – 0.3951	[43]
Getting Paxlovid or any other COVID medications	Point Estimate	0.25	-	[64]
Hospitalization for COVID-19 (unvaccinated)	Triangular	0.05	0.045 – 0.055	[65]
ICU admission for COVID-19, given hospitalization	Beta Pert	0.147	0.1323 – 0.1617	[55]
Mortality, COVID-19, given hospitalization[d]	Beta Pert	0.10	0.09 – 0.11	[55]
Hospitalization for:				
Hygiene-related infection (e.g., bacteremia, urinary tract infection [UTI], skin and soft tissue infection [SSTI], pneumonia)	Uniform	–	0.01 – 0.02	Expert Opinion, [5-7]
Mobility-related outcomes (e.g., falls, weakness, broken bones, pressure sores)	Uniform	–	0.01 – 0.03	Expert Opinion, [8-10]
Lack of feeding/hydration (e.g., dehydration, hypo-osomia, hypertension)	Uniform	–	0.02 – 0.06	Expert Opinion, [11,12]
Missed medication or medication errors	Uniform	–	0.01 – 0.02	Expert Opinion, [13,14]
In-hospital mortality due to[e]:				
Infection (e.g., bacteremia, UTI, SSTI, pneumonia)	Beta Pert	0.136	0.109 – 0.163	[26,66]
Mobility-related outcomes (e.g., falls, weakness, broken bones)	Beta Pert	0.015	0.012 – 0.019	[26,66]
Lack of feeding/hydration (e.g., dehydration, hypo-osomia, hypertension)	Beta Pert	0.024	0.019 – 0.029	[26,66]

© 2024 Bartsch SM et al. *JAMA Network Open*.

Missed medication or medication errors	Beta Pert	0.066	0.053 – 0.079	[26,66]
In-NH mortality due to negative outcomes/experiencing harm (daily)	Uniform	–	0.0011795 – 0.0012031	[67], Assumption[f]
**Costs (2024 US$)**				
Annual wages (all occupations; proxy for residents)[g]	Triangular	48,547	25,440 – 72,767	[58]
Over the counter medications, per dose	Gamma	0.108	0.417	[60]
Paxlovid	Point Estimate	1,668	-	[60]
Hospitalization for COVID-19[h]	Gamma	21,181.95	6,736.20 – 52,891.53	[68]
Nursing home physician visit for COVID-19 symptoms				
Initial	Uniform	–	133.52 – 182.31	[69]
Subsequent	Uniform	–	74.55 – 106.75	[69]
CBC w/ auto diff lab test	Point Estimate	7.77	–	[70]
Comprehensive metabolic panel lab test	Point Estimate	10.56	–	[70]
Cost per bed day for non-COVID-19 related hospitalizations	Triangular	3,533.96	3,478.04 – 3,589.88	[61]
**Durations (days)**				
Days until harm if task not completed				
Exercising/moving residents	Beta Pert	4	3 – 5	Expert Opinion, [8]
Turning (bed-bound residents)	Beta Pert	2	1 – 3	Expert Opinion,[9,10]
Feeding/hydrating	Beta Pert	2	1 – 3	Expert Opinion, [11,12]
Giving medications	Beta Pert	2.5	1 – 4	Expert Opinion, [13,14]
Providing toileting assistance or other hygiene related tasks	Beta Pert	2	1 – 3	Expert Opinion, [5-7]
Over the counter medication use (days) for COVID-19	Uniform	–	1 – 5	Assumption [i]
Duration of symptoms with mild COVID-19 (days)	Gamma	6.87	5.21	[54]
Duration of mild symptoms of COVID-19 prior to hospital admission	Beta Pert	7	3 – 9	[71,72]
Hospitalization for:				
COVID-19 (ICU and general ward)[j]	Gamma	3.9	1.9 – 8.7	[73]
Infection (e.g., bacteremia, UTI, SSTI, pneumonia)	Gamma	7.84	5.42	[26,66]
Mobility-related outcomes (e.g., falls, weakness, broken bones)	Gamma	7.5	7.0	[26,66]
Lack of feeding/hydration (e.g., dehydration, hypo-osomia, hypertension)	Gamma	6.04	3.69	[26,66]
Missed medication or medication errors	Gamma	7.21	8.56	[26,66]
**Numbers**				

© 2024 Bartsch SM et al. *JAMA Network Open*.

Over the counter medication doses per day	Uniform	–	4 – 6	Standard dosing
Number of times per day an isolated resident is visited by staff wearing PPE[k]	Point	50		74
**Utility weights**				
Healthy QALY ≥65 years old	Point Estimate	0.84	–	62
Mild non-specific COVID-19 symptoms	Beta	0.8179	0.1210	75-85
Severe COVID-19 infection, leading to hospitalization	Beta	0.489	0.209	81,86-93
Infection (e.g., bacteremia, UTI, SSTI, pneumonia)	Beta	0.6291	0.0857	92,94-101
Mobility-related outcomes (e.g., falls, weakness, broken bones)	Beta	0.6268	0.0698	102-108
Lack of feeding/hydration (e.g., dehydration, hypo-osomia, hypertension)	Beta	0.5575	0.2406	109-114
Missed medication or medication errors	Beta	0.6492	0.1864	88,115-123

[a] Bathing tasks occur 3x per week; not all residents are bathed every day.
[b] Includes 20 bed bound and 30 mobile residents.
[c] Value calibrated such that of those showing symptoms, 75% are due to COVID-19 and the remaining 25% are due to other respiratory pathogens during a respiratory virus season.
[d] Values are +/-10% of median/mean value.
[e] Values are +/-20% of likeliest value.
[f] Assumed to be 0 – 2% higher than natural rate.
[g] Values are median, 10th and 90th percentiles.
[h] Average Medicare payment per fee-for-service COVID-19 hospitalization reported between January and June 2022.
[i] Based on symptom duration.
[j] Values are median, interquartile range (IQR).
[k] Estimated isolated residents visited by staff 4.7x per hour (total 11 hours).

© 2024 Bartsch SM et al. *JAMA Network Open*.

eFigure 3. Impact of Furloughing Staff Testing Positive for COVID-19 When Assuming Greater Virus Transmissibility (Probability of Transmission 0.1 per Contact) on the Average Number of (A) Missed Resident Care Tasks, (B) Resident Hospitalizations, (C) Resident Deaths, (D) Costs Incurred From the CMS Perspective, and (E) Costs Incurred From the Societal Perspective
Note difference in axis scales across panels.

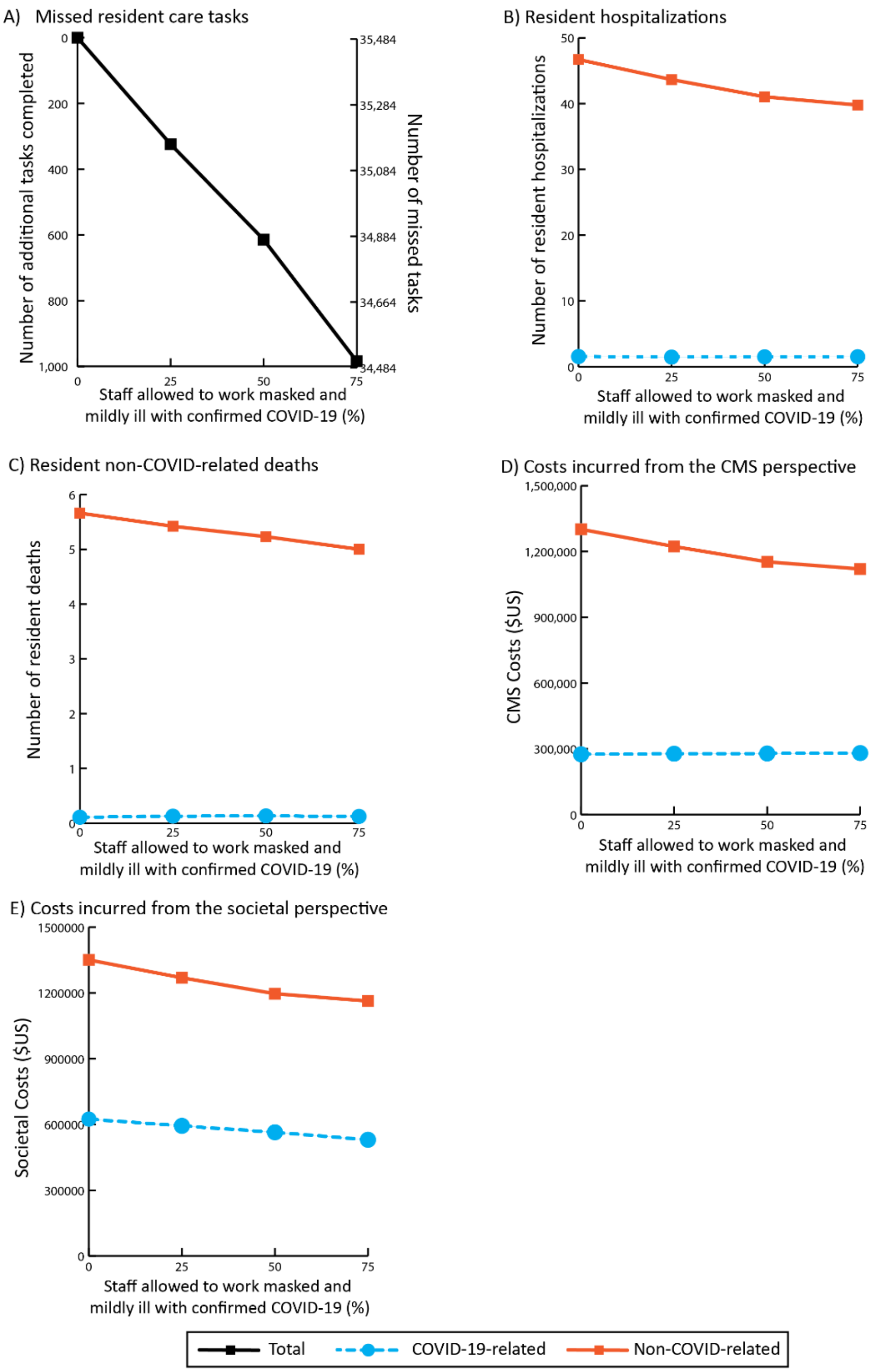

© 2024 Bartsch SM et al. *JAMA Network Open*.

eReferences

1. Bartsch SM, Weatherwax C, Martinez MF, et al. Cost-effectiveness of severe acute respiratory coronavirus virus 2 (SARS-CoV-2) testing and isolation strategies in nursing homes. *Infect Control Hosp Epidemiol.* 2024:1-8.
2. Bartsch SM, Weatherwax C, Wasserman MR, et al. How the Timing of Annual COVID-19 Vaccination of Nursing Home Residents and Staff Affects Its Value. *J Am Med Dir Assoc.* 2024.
3. Stein C, Nassereldine H, Sorensen RJD, et al. Past SARS-CoV-2 infection protection against re-infection: a systematic review and meta-analysis. *The Lancet (British edition).* 2023;401(10379):833-842.
4. Human Morality Database. University of California, Berkeley (USA), and Max Planck Institute for Demographic Research (Germany); 2015. www.mortality.org. Accessed December 20, 2017.
5. Rosenblum R, Jr. Oral hygiene can reduce the incidence of and death resulting from pneumonia and respiratory tract infection. *J Am Dent Assoc.* 2010;141(9):1117-1118.
6. Andersson A, Frank C, Willman AM, Sandman PO, Hansebo G. Factors contributing to serious adverse events in nursing homes. *J Clin Nurs.* 2018;27(1-2):e354-e362.
7. Grimsland F, Seim A, Borza T, Helvik AS. Toileting difficulties in older people with and without dementia receiving formal in-home care-A longitudinal study. *Nurs Open.* 2019;6(3):1055-1066.
8. English KL, Paddon-Jones D. Protecting muscle mass and function in older adults during bed rest. *Curr Opin Clin Nutr Metab Care.* 2010;13(1):34-39.
9. Gefen A. How much time does it take to get a pressure ulcer? Integrated evidence from human, animal, and in vitro studies. *Ostomy Wound Manage.* 2008;54(10):26-28, 30-25.
10. Asiri S. Turning and Repositioning Frequency to Prevent Hospital-Acquired Pressure Injuries Among Adult Patients: Systematic Review. *Inquiry.* 2023;60:469580231215209.
11. Hooper L, Abdelhamid A, Attreed NJ, et al. Clinical symptoms, signs and tests for identification of impending and current water-loss dehydration in older people. *Cochrane Database Syst Rev.* 2015;2015(4):CD009647.
12. Brunner S, Mayer H, Qin H, Breidert M, Dietrich M, Muller Staub M. Interventions to optimise nutrition in older people in hospitals and long-term care: Umbrella review. *Scand J Caring Sci.* 2022;36(3):579-598.
13. Barber ND, Alldred DP, Raynor DK, et al. Care homes' use of medicines study: prevalence, causes and potential harm of medication errors in care homes for older people. *Qual Saf Health Care.* 2009;18(5):341-346.
14. Gurwitz JH, Field TS, Avorn J, et al. Incidence and preventability of adverse drug events in nursing homes. *Am J Med.* 2000;109(2):87-94.
15. Maryland Department of Health. Title 10: Maryland Department of Health Regulations. Subtitle 07: Hospitals 10.07.02 [Comprehensive Care Facilities and Extended Care Facilities] Nursing Homes. In. Baltimore, MD: Maryland Department of Health; 2019.
16. Mukamel DB, Saliba D, Ladd H, Konetzka RT. Daily Variation in Nursing Home Staffing and Its Association With Quality Measures. *JAMA Netw Open.* 2022;5(3):e222051.
17. Kane RL, Huckfeldt P, Tappen R, et al. Effects of an Intervention to Reduce Hospitalizations From Nursing Homes: A Randomized Implementation Trial of the INTERACT Program. *JAMA Intern Med.* 2017;177(9):1257-1264.
18. Schnelle JF, Schroyer LD, Saraf AA, Simmons SF. Determining Nurse Aide Staffing Requirements to Provide Care Based on Resident Workload: A Discrete Event Simulation Model. *J Am Med Dir Assoc.* 2016;17(11):970-977.
19. Nelson ST, Flynn L. Relationship between missed care and urinary tract infections in nursing homes. *Geriatr Nurs.* 2015;36(2):126-130.
20. Hackman P, Hult M, Haggman-Laitila A. Unfinished nursing care in nursing homes. *Geriatr Nurs.* 2023;51:33-39.
21. Campagna S, Basso I, Vercelli E, et al. Missed Nursing Care in a Sample of High-Dependency Italian Nursing Home Residents: Description of Nursing Care in Action. *J Patient Saf.* 2021;17(8):e1840-e1845.
22. Spector WD, Limcangco R, Williams C, Rhodes W, Hurd D. Potentially avoidable hospitalizations for elderly long-stay residents in nursing homes. *Med Care.* 2013;51(8):673-681.
23. Centers for Disease Control and Prevention. Nursing Home Covid-19 Data Dashboard. U.S. Department of Health & Human Services. https://www.cdc.gov/nhsn/covid19/ltc-report-overview.html. Updated March 30, 2023. Accessed June 14, 2024.
24. Franklin D, Barbre K, Rowe TA, et al. COVID-19 Vaccination Coverage, and Rates of SARS-CoV-2 Infection and COVID-19-Associated Hospitalization Among Residents in Nursing Homes - National

© 2024 Bartsch SM et al. *JAMA Network Open*.

Healthcare Safety Network, United States, October 2023-February 2024. *MMWR Morb Mortal Wkly Rep.* 2024;73(15):339-344.
25. Office of Statewide Health Planning and Development. *California Inpatient Data Reporting Manual, Medical Information Reporting for California, Seventh Edition, Version 8.3 2014.* 2014.
26. Centers for Medicare & Medicaid Services (CMS). Minimum Data Set (MDS) 3.0 Frequency Report. In:2019.
27. Gussin GM, McKinnell JA, Singh RD, et al. Reducing Hospitalizations and Multidrug-Resistant Organisms via Regional Decolonization in Hospitals and Nursing Homes. *The Journal of the American Medical Association.* 2024.
28. Wu Y, Kang L, Guo Z, Liu J, Liu M, Liang W. Incubation Period of COVID-19 Caused by Unique SARS-CoV-2 Strains: A Systematic Review and Meta-analysis. *JAMA Netw Open.* 2022;5(8):e2228008.
29. Kim D, Ali ST, Kim S, et al. Estimation of Serial Interval and Reproduction Number to Quantify the Transmissibility of SARS-CoV-2 Omicron Variant in South Korea. *Viruses.* 2022;14(3).
30. Backer JA, Eggink D, Andeweg SP, et al. Shorter serial intervals in SARS-CoV-2 cases with Omicron BA.1 variant compared with Delta variant, the Netherlands, 13 to 26 December 2021. *Euro Surveill.* 2022;27(6).
31. Del Aguila-Mejia J, Wallmann R, Calvo-Montes J, Rodriguez-Lozano J, Valle-Madrazo T, Aginagalde-Llorente A. Secondary Attack Rate, Transmission and Incubation Periods, and Serial Interval of SARS-CoV-2 Omicron Variant, Spain. *Emerg Infect Dis.* 2022;28(6):1224-1228.
32. Centers for Disease Control and Prevention. CDC Updates and Shortens Recommended Isolation and Quarantine Period for General Population. https://www.cdc.gov/media/releases/2021/s1227-isolation-quarantine-guidance.html. Published 2022. Accessed February 15, 2023.
33. Boucau J, Marino C, Regan J, et al. Duration of Shedding of Culturable Virus in SARS-CoV-2 Omicron (BA.1) Infection. *N Engl J Med.* 2022;387(3):275-277.
34. Centers for Disease Control and Prevention. Vaccination Coverage among Nursing Home Residents.
35. Hansen CH, Moustsen-Helms IR, Rasmussen M, Soborg B, Ullum H, Valentiner-Branth P. Short-term effectiveness of the XBB.1.5 updated COVID-19 vaccine against hospitalisation in Denmark: a national cohort study. *Lancet Infect Dis.* 2024;24(2):e73-e74.
36. Centers for Disease Control and Prevention. Science Brief: SARS-CoV-2 Infection-induced and Vaccine-induced Immunity. https://www.cdc.gov/coronavirus/2019-ncov/science/science-briefs/vaccine-induced-immunity.html. Updated October 29, 2021. Accessed July 31, 2023.
37. Stein C, Nassereldine H, Sorensen RJD, et al. Past SARS-CoV-2 infection protection against re-infection: a systematic review and meta-analysis. *The Lancet.* 2023;401(10379):833-842.
38. Lindsley WG, Blachere FM, Law BF, Beezhold DH. Efficacy of face masks, neck gaiters and face shields for reducing the expulsion of simulated cough-generated aerosols. *Aerosol Science and Technology.* 2021;55(4):449-457.
39. Kendra MA, Weiker A, Simon S, Grant A, Shullick D. Safety concerns affecting delivery of home health care. *Public Health Nurs.* 1996;13(2):83-89.
40. Hayden MK, Mustafa RA, Hanson KE, et al. Infectious Diseases Society of America Guidelines on the Diagnosis of COVID-19: Antigen Testing. *Infectious Disease Society of America.* 2022.
41. Hanson KE, Caliendo AM, Arias CA, et al. Infectious Diseases Society of America Guidelines on the Diagnosis of COVID-19: Molecular Diagnostic Testing. *Infectious Disease Society of America.* 2022.
42. Gussin GM, Singh RD, Tjoa TT, Saavedra R, Kaplan SH, Huang SS. Evaluating barriers and potential solutions to speaking up about coronavirus disease 2019 (COVID-19) symptoms: A survey among nursing home workers. *Infect Control Hosp Epidemiol.* 2023;44(11):1834-1839.
43. Shang W, Kang L, Cao G, et al. Percentage of Asymptomatic Infections among SARS-CoV-2 Omicron Variant-Positive Individuals: A Systematic Review and Meta-Analysis. *Vaccines (Basel).* 2022;10(7).
44. Centers for Disease Control and Prevention (CDC). Interim Guidance for Managing Healthcare Personnel with SARS-CoV-2 Infection or Exposure to SARS-CoV-2. Centers for Disease Control and Prevention. https://www.cdc.gov/coronavirus/2019-ncov/hcp/guidance-risk-assesment-hcp.html. Published 2022. Updated September 23, 2022. Accessed December 12, 2023.
45. Dawson L, Amin K, Kates J, Cox C. How Are Private Insurers Covering At-Home Rapid COVID Tests? Kaiser Family Foundation. https://www.kff.org/policy-watch/how-are-private-insurers-covering-at-home-rapid-covid-tests/. Published 2022. Accessed February 6, 2023.
46. 3M. Get the Facts. N95 Respirator Pricing. https://multimedia.3m.com/mws/media/1862179O/get-the-facts-n95-respirator-pricing.pdf. Published 2021. Accessed.

© 2024 Bartsch SM et al. *JAMA Network Open*.

47. World Health Organization. WHO COVID-19 Essential Supplies Forecasting Tool (COVID-ESFT) v4.1. https://www.who.int/publications/i/item/WHO-2019-nCoV-Tools-Essential_forecasting-2022.1. Published 2022. Accessed January 29,, 2024.
48. Clinical Supply Co. Posi-Prene Powder/Latex-Free Exam Gloves, White, 100 gloves per box. https://clinicalsupplycompany.com/collections/posi-prene/products/white-posi-prene-gloves-powder-free. Published 2024. Accessed January 29,, 2024.
49. ULINE. Uline Isolation Gowns - Blue. https://www.uline.com/Product/Detail/S-24019BLU/Disposable-Clothing/Uline-Isolation-Gowns-Blue. Published 2024. Accessed January 29,, 2024.
50. Duraline Biosystems Inc. Full Face Shield with Foam Forehead Band and Elastic Strap, Anti Fog 10 per pack. https://www.duralinesystems.com/Face-Shields-with-Padding-p/c-fs-pk10.htm. Published 2024. Accessed January 28,, 2024.
51. Gandhi A, Yu H, Grabowski DC. High Nursing Staff Turnover In Nursing Homes Offers Important Quality Information. *Health Affairs.* 2021;40(3).
52. Network H-R, Thompson MG, Yoon SK, et al. Association of mRNA Vaccination With Clinical and Virologic Features of COVID-19 Among US Essential and Frontline Workers. *JAMA.* 2022;328(15):1523-1533.
53. Shah MM, Joyce B, Plumb ID, et al. Paxlovid Associated with Decreased Hospitalization Rate Among Adults with COVID-19 - United States, April-September 2022. *MMWR Morbidity and mortality weekly report.* 2022;71(48):1531-1537.
54. Menni C, Valdes AM, Polidori L, et al. Symptom prevalence, duration, and risk of hospital admission in individuals infected with SARS-CoV-2 during periods of omicron and delta variant dominance: a prospective observational study from the ZOE COVID Study. *Lancet.* 2022;399(10335):1618-1624.
55. Iuliano AD, Brunkard JM, Boehmer TK, et al. Trends in Disease Severity and Health Care Utilization During the Early Omicron Variant Period Compared with Previous SARS-CoV-2 High Transmission Periods - United States, December 2020-January 2022. *MMWR Morb Mortal Wkly Rep.* 2022;71(4):146-152.
56. Knopp-Sihota JA, Niehaus L, Squires JE, Norton PG, Estabrooks CA. Factors associated with rushed and missed resident care in western Canadian nursing homes: a cross-sectional survey of health care aides. *J Clin Nurs.* 2015;24(19-20):2815-2825.
57. Song Y, Hoben M, Norton P, Estabrooks CA. Association of Work Environment With Missed and Rushed Care Tasks Among Care Aides in Nursing Homes. *JAMA Netw Open.* 2020;3(1):e1920092.
58. U.S. Bureau of Labor Statistics. Occupational Employment and Wage Statistics. U.S. Bureau of Labor Statistics. https://www.bls.gov/oes/tables.htm. Published 2021. Updated March 31, 2022. Accessed October 27, 2022.
59. Centers for Medicare & Medicaid Services. Physicians Fee Schedule. U.S. Centers for Medicare & Medicaid Services. https://www.cms.gov/medicare/physician-fee-schedule/search/overview. Published 2022. Updated January 1, 2022. Accessed March 10, 2022.
60. IBM. Micromedex RED BOOK. 2022.
61. https://datatools.ahrq.gov/hcupnet. Agency for Healthcare Research and Quality; 2020. https://datatools.ahrq.gov/hcupnet.
62. Gold MR, Franks P, McCoy KI, Fryback DG. Toward consistency in cost-utility analyses: using national measures to create condition-specific values. *Medical Care.* 1998;36(6):778-792.
63. Harrington C, Edelman TS. Failure to Meet Nurse Staffing Standards: A Litigation Case Study of a Large US Nursing Home Chain. *Inquiry.* 2018;55:46958018788686.
64. McGarry BE, Sommers BD, Wilcock AD, Grabowski DC, Barnett ML. Monoclonal Antibody and Oral Antiviral Treatment of SARS-CoV-2 Infection in US Nursing Homes. *JAMA.* 2023;330(6):561-563.
65. Esper FP, Adhikari TM, Tu ZJ, et al. Alpha to Omicron: Disease Severity and Clinical Outcomes of Major SARS-CoV-2 Variants. *J Infect Dis.* 2023;227(3):344-352.
66. Centers for Medicare & Medicaid Services (CMS). Research Data Assistance Center (ResDAC). https://resdac.org/. Published 2024. Accessed.
67. Li S, Middleton A, Ottenbacher KJ, Goodwin JS. Trajectories Over the First Year of Long-Term Care Nursing Home Residence. *J Am Med Dir Assoc.* 2018;19(4):333-341.
68. Centers for Medicare & Medicaid Services (CMS). *Medicare COVID-19 Hospitalization Trends Report: Medicare Claims and Encounter Data: January 1, 2020 to June 30, 2022, Received by October 28, 2022.* CMS.gov;2022.

© 2024 Bartsch SM et al. *JAMA Network Open*.

69. Centers for Medicare & Medicaid Services (CMS). Physician Fee Schedule. Centers for Medicare & Medicaid Services. https://www.cms.gov/apps/physician-fee-schedule/. Published 2023. Updated April 3, 2023. Accessed June 21, 2023.
70. Centers for Medicare & Medicaid Services (CMS). Clinical Laboratory Fee Schedule Files. https://www.cms.gov/Medicare/Medicare-Fee-for-Service-Payment/ClinicalLabFeeSched/Clinical-Laboratory-Fee-Schedule-Files. Published 2023. Accessed February 6, 2023.
71. Garg S, Kim L, Whitaker M, et al. Hospitalization Rates and Characteristics of Patients Hospitalized with Laboratory-Confirmed Coronavirus Disease 2019 — COVID-NET, 14 States, March 1–30, 2020. *Morbidity and Mortality Weekly Report.* ePub: 8 April 2020.
72. Wang D, Hu B, Hu C, et al. Clinical Characteristics of 138 Hospitalized Patients With 2019 Novel Coronavirus-Infected Pneumonia in Wuhan, China. *JAMA.* 2020.
73. Havers FP, Patel K, Whitaker M, et al. Laboratory-Confirmed COVID-19-Associated Hospitalizations Among Adults During SARS-CoV-2 Omicron BA.2 Variant Predominance - COVID-19-Associated Hospitalization Surveillance Network, 14 States, June 20, 2021-May 31, 2022. *MMWR Morb Mortal Wkly Rep.* 2022;71(34):1085-1091.
74. Pineles L, Petruccelli C, Perencevich EN, et al. The Impact of Isolation on Healthcare Worker Contact and Compliance With Infection Control Practices in Nursing Homes. *Infect Control Hosp Epidemiol.* 2018;39(6):683-687.
75. Chen D, Ye Z, Pi Z, Mizukami S, Aoyagi K, Jiang Y. Cost-effectiveness of dual influenza and pneumococcal vaccination among the elderly in Shenzhen, China. *Vaccine.* 2021;39(16):2237-2245.
76. Griffin AD, Perry AS, Fleming DM. Cost-effectiveness analysis of inhaled zanamivir in the treatment of influenza A and B in high-risk patients. *Pharmacoeconomics.* 2001;19(3):293-301.
77. Lee GM, Murphy TV, Lett S, et al. Cost effectiveness of pertussis vaccination in adults. *Am J Prev Med.* 2007;32(3):186-193.
78. Lee GM, Riffelmann M, Wirsing von Konig CH. Cost-effectiveness of adult pertussis vaccination in Germany. *Vaccine.* 2008;26(29-30):3673-3679.
79. Mennini FS, Bini C, Marcellusi A, Rinaldi A, Franco E. Cost-effectiveness of switching from trivalent to quadrivalent inactivated influenza vaccines for the at-risk population in Italy. *Hum Vaccin Immunother.* 2018;14(8):1867-1873.
80. Scholz SM, Weidemann F, Damm O, Ultsch B, Greiner W, Wichmann O. Cost-Effectiveness of Routine Childhood Vaccination Against Seasonal Influenza in Germany. *Value Health.* 2021;24(1):32-40.
81. Wu DBC, Chaiyakunapruk N, Pratoomsoot C, et al. Cost-utility analysis of antiviral use under pandemic influenza using a novel approach - linking pharmacology, epidemiology and heath economics. *Epidemiol Infect.* 2018;146(4):496-507.
82. Khazeni N, Hutton DW, Garber AM, Hupert N, Owens DK. Effectiveness and cost-effectiveness of vaccination against pandemic influenza (H1N1) 2009. *Ann Intern Med.* 2009;151(12):829-839.
83. Lee BY, Tai JHY, Bailey RR, Smith KJ, Nowalk AJ. Economics of influenza vaccine administration timing for children. *American Journal of Managed Care.* 2010;16(3):e75-e85.
84. Smith KJ, Lee BY, Nowalk MP, Raymund M, Zimmerman RK. Cost-effectiveness of dual influenza and pneumococcal vaccination in 50-year-olds. *Vaccine.* 2010;28:7620-7625.
85. Prosser LA, Meltzer MI, Fiore A, et al. Effects of adverse events on the projected population benefits and cost-effectiveness of using live attenuated influenza vaccine in children aged 6 months to 4 years. *Arch Pediatr Adolesc Med.* 2011;165(2):112-118.
86. Elliott RA, Putman KD, Franklin M, et al. Cost effectiveness of a pharmacist-led information technology intervention for reducing rates of clinically important errors in medicines management in general practices (PINCER). *Pharmacoeconomics.* 2014;32(6):573-590.
87. Mohara A, Perez Velasco R, Praditsitthikorn N, Avihingsanon Y, Teerawattananon Y. A cost-utility analysis of alternative drug regimens for newly diagnosed severe lupus nephritis patients in Thailand. *Rheumatology (Oxford).* 2014;53(1):138-144.
88. Chandra A, Snider JT, Wu Y, Jena A, Goldman DP. Robot-assisted surgery for kidney cancer increased access to a procedure that can reduce mortality and renal failure. *Health Aff (Millwood).* 2015;34(2):220-228.
89. Fuller GW, Keating S, Goodacre S, et al. Prehospital continuous positive airway pressure for acute respiratory failure: the ACUTE feasibility RCT. *Health Technol Assess.* 2021;25(7):1-92.
90. Cox CE, Carson SS, Biddle AK. Cost-effectiveness of ultrasound in preventing femoral venous catheter-associated pulmonary embolism. *Am J Respir Crit Care Med.* 2003;168(12):1481-1487.

© 2024 Bartsch SM et al. *JAMA Network Open*.

91. Kip MMA, van Oers JA, Shajiei A, et al. Cost-effectiveness of procalcitonin testing to guide antibiotic treatment duration in critically ill patients: results from a randomised controlled multicentre trial in the Netherlands. *Crit Care.* 2018;22(1):293.
92. Fowler RA, Hill-Popper M, Stasinos J, Petrou C, Sanders GD, Garber AM. Cost-effectiveness of recombinant human activated protein C and the influence of severity of illness in the treatment of patients with severe sepsis. *J Crit Care.* 2003;18(3):181-191; discussion 191-184.
93. Kotirum S, Muangchana C, Techathawat S, Dilokthornsakul P, Wu DB, Chaiyakunapruk N. Economic Evaluation and Budget Impact Analysis of Vaccination against Haemophilus influenzae Type b Infection in Thailand. *Front Public Health.* 2017;5:289.
94. Wang R, LaSala C. Role of antibiotic resistance in urinary tract infection management: a cost-effectiveness analysis. *Am J Obstet Gynecol.* 2021;225(5):550 e551-550 e510.
95. Talmor D, Greenberg D, Howell MD, Lisbon A, Novack V, Shapiro N. The costs and cost-effectiveness of an integrated sepsis treatment protocol. *Crit Care Med.* 2008;36(4):1168-1174.
96. Slobogean GP, O'Brien PJ, Brauer CA. Single-dose versus multiple-dose antibiotic prophylaxis for the surgical treatment of closed fractures. *Acta Orthop.* 2010;81(2):256-262.
97. Simon MS, Sfeir MM, Calfee DP, Satlin MJ. Cost-effectiveness of ceftazidime-avibactam for treatment of carbapenem-resistant Enterobacteriaceae bacteremia and pneumonia. *Antimicrob Agents Chemother.* 2019;63(12).
98. Lee GM, Lebaron C, Murphy TV, Lett S, Schauer S, Lieu TA. Pertussis in adolescents and adults: should we vaccinate? *Pediatrics.* 2005;115(6):1675-1684.
99. Lee BY, Wiringa AE, Bailey RR, Lewis GJ, Feura J, Muder RR. Staphylococcus aureus vaccine for orthopedic patients: an economic model and analysis. *Vaccine.* 2010;28(12):2465-2471.
100. Chopra K, Gowda AU, Morrow C, Holton L, 3rd, Singh DP. The Economic Impact of Closed-Incision Negative-Pressure Therapy in High-Risk Abdominal Incisions: A Cost-Utility Analysis. *Plast Reconstr Surg.* 2016;137(4):1284-1289.
101. Bermingham SL, Hodgkinson S, Wright S, Hayter E, Spinks J, Pellowe C. Intermittent self catheterisation with hydrophilic, gel reservoir, and non-coated catheters: a systematic review and cost effectiveness analysis. *BMJ.* 2013;346:e8639.
102. Williams J, Roberts I, Shakur-Still H, Lecky FE, Chaudhri R, Miners A. Cost-effectiveness analysis of tranexamic acid for the treatment of traumatic brain injury, based on the results of the CRASH-3 randomised trial: a decision modelling approach. *BMJ Glob Health.* 2020;5(9).
103. Skou ST, Koes BW, Gronne DT, Young J, Roos EM. Comparison of three sets of clinical classification criteria for knee osteoarthritis: a cross-sectional study of 13,459 patients treated in primary care. *Osteoarthritis Cartilage.* 2020;28(2):167-172.
104. Premkumar A, Lebrun DG, Sidharthan S, et al. Bariatric Surgery Prior to Total Hip Arthroplasty Is Cost-Effective in Morbidly Obese Patients. *J Arthroplasty.* 2020;35(7):1766-1775 e1763.
105. Karnon J, Afzali HHA, Putro G, et al. A Cost-Effectiveness Model for Frail Older Persons: Development and Application to a Physiotherapy-Based Intervention. *Appl Health Econ Health Policy.* 2017;15(5):635-645.
106. Johnell O, Jonsson B, Jonsson L, Black D. Cost effectiveness of alendronate (fosamax) for the treatment of osteoporosis and prevention of fractures. *Pharmacoeconomics.* 2003;21(5):305-314.
107. Jensen MP, Tome-Pires C, de la Vega R, Galan S, Sole E, Miro J. What Determines Whether a Pain is Rated as Mild, Moderate, or Severe? The Importance of Pain Beliefs and Pain Interference. *Clin J Pain.* 2017;33(5):414-421.
108. Friedman J, Reed P, Sharplin P, Kelly P. Primary prevention of pediatric abusive head trauma: a cost audit and cost-utility analysis. *Child Abuse Negl.* 2012;36(11-12):760-770.
109. Djalalov S, Beca J, Amir E, Krahn M, Trudeau ME, Hoch JS. Economic evaluation of hormonal therapies for postmenopausal women with estrogen receptor-positive early breast cancer in Canada. *Curr Oncol.* 2015;22(2):84-96.
110. Critchley DJ, Ratcliffe J, Noonan S, Jones RH, Hurley MV. Effectiveness and cost-effectiveness of three types of physiotherapy used to reduce chronic low back pain disability: a pragmatic randomized trial with economic evaluation. *Spine (Phila Pa 1976).* 2007;32(14):1474-1481.
111. Ackerman SJ, Deol GS, Polly DW. Cost-Utility Analysis of Sacroiliac Joint Fusion in High-Risk Patients Undergoing Multi-Level Lumbar Fusion to the Sacrum. *Clinicoecon Outcomes Res.* 2022;14:523-535.

© 2024 Bartsch SM et al. *JAMA Network Open*.

112. Zhong Y, Cohen JT, Goates S, Luo M, Nelson J, Neumann PJ. The Cost-Effectiveness of Oral Nutrition Supplementation for Malnourished Older Hospital Patients. *Appl Health Econ Health Policy.* 2017;15(1):75-83.
113. Park SK, Hong SH, Kim H, Kim S, Lee EK. Cost-Utility Analysis of Sacubitril/Valsartan Use Compared With Standard Care in Chronic Heart Failure Patients With Reduced Ejection Fraction in South Korea. *Clin Ther.* 2019;41(6):1066-1079.
114. Mosegui GG, Vianna CM, Rodrigues MS, Valle PM, Silva FV. Cost-effectiveness analysis of oral rehydration therapy compared to intravenous rehydration for acute gastroenteritis without severe dehydration treatment. *J Infect Public Health.* 2019;12(6):816-821.
115. Tisdale RL, Cusick MM, Aluri KZ, et al. Cost-Effectiveness of Dapagliflozin for Non-diabetic Chronic Kidney Disease. *J Gen Intern Med.* 2022;37(13):3380-3387.
116. Sobocki P, Ekman M, Ovanfors A, Khandker R, Jonsson B. The cost-utility of maintenance treatment with venlafaxine in patients with recurrent major depressive disorder. *Int J Clin Pract.* 2008;62(4):623-632.
117. Rajagopalan K, Trueman D, Crowe L, Squirrell D, Loebel A. Cost-Utility Analysis of Lurasidone Versus Aripiprazole in Adults with Schizophrenia. *Pharmacoeconomics.* 2016;34(7):709-721.
118. Johnson KM, Sadatsafavi M, Adibi A, et al. Cost Effectiveness of Case Detection Strategies for the Early Detection of COPD. *Appl Health Econ Health Policy.* 2021;19(2):203-215.
119. Hornberger J, Li Q, Quinn B. Cost-effectiveness of combinatorial pharmacogenomic testing for treatment-resistant major depressive disorder patients. *Am J Manag Care.* 2015;21(6):e357-365.
120. Carlos RC, Axelrod DA, Ellis JH, Abrahamse PH, Fendrick AM. Incorporating patient-centered outcomes in the analysis of cost-effectiveness: imaging strategies for renovascular hypertension. *AJR Am J Roentgenol.* 2003;181(6):1653-1661.
121. Cameron CG, Bennett HA. Cost-effectiveness of insulin analogues for diabetes mellitus. *CMAJ.* 2009;180(4):400-407.
122. Andrews G, Sanderson K, Corry J, Issakidis C, Lapsley H. Cost-effectiveness of current and optimal treatment for schizophrenia. *Br J Psychiatry.* 2003;183:427-435; discussion 436.
123. Aberg F, Maklin S, Rasanen P, et al. Cost of a quality-adjusted life year in liver transplantation: the influence of the indication and the model for end-stage liver disease score. *Liver Transpl.* 2011;17(11):1333-1343.

© 2024 Bartsch SM et al. *JAMA Network Open*.
