# Supplementary material for: Modeling Nursing Home Harms From COVID-19 Staff Furlough Policies
Source: JAMA Netw Open. 2024 Aug 19;7(8):e2429613. doi: 10.1001/jamanetworkopen.2024.29613 (PMC11333984; doi:10.1001/jamanetworkopen.2024.29613)
Supplement: Supplement 2. — Data Sharing Statement [file jamanetwopen-e2429613-s002.pdf]

Data Sharing Statement

Bartsch. Modeling Nursing Home Harms From COVID-19 Staff Furlough Policies. *JAMA Netw Open*. Published August 22, 2024. doi:10.1001/jamanetworkopen.2024.29613

Data
Data available: Yes
Data types: Data (not involving human participants)
How to access data: All data are provided in the Appendix.
When available: With publication

Supporting Documents
Document types: None

Additional Information
Who can access the data: All data are contained within the publication
Types of analyses: NA
Mechanisms of data availability: All data are contained within the publication
